# Methylation of BRD4 by PRMT1 regulates BRD4 phosphorylation and promotes ovarian cancer invasion

**DOI:** 10.1038/s41419-023-06149-5

**Published:** 2023-09-22

**Authors:** Yi Liu, Hejing Liu, Miaomiao Ye, Mengying Jiang, Xin Chen, Gendi Song, Huihui Ji, Zhi-wei Wang, Xueqiong Zhu

**Affiliations:** 1 Zhejiang Provincial Clinical Research Center for Obstetrics and Gynecology, Department of Obstetrics and Gynecology, the Second Affiliated Hospital of Wenzhou Medical University, 325027 Wenzhou, China; 2 Department of Pathology, Beth Israel Deaconess Medical Center, Harvard Medical School, Boston, MA USA

**Keywords:** Ovarian cancer, Oncogenes

## Abstract

Bromodomain-containing protein 4 (BRD4), the major component of bromodomain and extra-terminal domain (BET) protein family, has important functions in early embryonic development and cancer development. However, the posttranslational modification of BRD4 is not well understood. Multiple approaches were used to explore the mechanism of PRMT1-mediated BRD4 methylation and to determine the biological functions of BRD4 and PRMT1 in ovarian cancer. Here we report that BRD4 is asymmetrically methylated at R179/181/183 by PRMT1, which is antagonized by the Jumonji-family demethylase, JMJD6. PRMT1 is overexpressed in ovarian cancer tissue and is a potential marker for poor prognosis in ovarian cancer patients. Silencing of PRMT1 inhibited ovarian cancer proliferation, migration, and invasion in vivo and in vitro. PRMT1-mediated BRD4 methylation was found to promote BRD4 phosphorylation. Compared to BRD4 wild-type (WT) cells, BRD4 R179/181/183K mutant-expressing cells showed reduced ovarian cancer metastasis. BRD4 arginine methylation is also associated with TGF-β signaling. Our results indicate that arginine methylation of BRD4 by PRMT1 is involved in ovarian cancer tumorigenesis. Targeting PRMT1-mediated arginine methylation may provide a novel diagnostic target and an effective therapeutic strategy for ovarian cancer treatment.

## Introduction

Bromodomain-containing protein 4 (BRD4) belongs to the bromodomain and extra-terminal domain (BET) protein family, which also contains BRD2, BRD3, and BRDT. BRD4, an epigenetic and transcriptional regulator, plays a critical role in early embryonic development, tumorigenesis, and cancer progression [1, 2]. BRD4 is characterized by two N-terminal bromodomain domains (BD1 and BD2) that bind to acetylated lysine residues on target proteins, a structurally defined ET domain and a C-terminal domain (CTD) [3–5]. Emerging evidence has illustrated that BRD4 is involved in the development of many cancer types, such as pancreatic cancer [6], colorectal cancer [7], glioblastoma [8], and ovarian cancer [9]. BRD4 regulates cancer cell migration and invasion and is associated with poor clinical outcomes [10, 11]. BRD4 has been proposed as a druggable promising target in human cancer [12–14].

Several studies have revealed that post-translational modifications (PTMs) of BRD4 are pivotal to its biological function. Janus kinase 2 (JAK2)-mediated BRD4 phosphorylation at tyrosine 97 and tyrosine 98 induces protein stabilization [7]. Besides the phosphorylation, the speckle type BTB/POZ protein (SPOP)-mediated BRD4 ubiquitination regulates BRD4 degradation in prostate cancer [15]. A recent study reported that BRD4 methylation by SET domain containing 6 (SETD6) at K99 regulates and controls mRNA translation [16]. BRD4 upstream regulation by PTMs, however, remains largely unknown.

Protein arginine methylation has been identified as one of the most common PTMs, resulting in monomethylarginine (MMA), asymmetrical dimethylarginine (ADMA), or symmetrical dimethylarginine (SDMA) [17]. Protein arginine methylation is catalyzed by a family of enzymes termed protein arginine methyltransferases (PRMTs), including nine PRMT family members. Among these nine PRMTs, PRMTs 1, 2, 3, 4, 6, and 8 are characterized as type I enzymes that generate ADMA modification, whereas PRMT5 and PRMT9 are identified as type II enzymes that promote SDMA, and type III enzyme (PRMT7) only forms MMA [17]. These PRMTs are involved in many essential cellular processes, including DNA replication, transcription, RNA splicing, and protein degradation by arginine methylation of targeted proteins [18, 19]. Deregulation of PRMTs participates in carcinogenesis, metabolism, immune, neurodegenerative, and muscular disorders [20–22].

PRMT1, a main type I enzyme, is responsible for the majority of ADMA formation in mammals [23]. A growing number of studies indicated that ADMA modification by PRMT1 participates in various biological processes, including tumorigenesis and cancer metastasis [24–28]. Overexpression of PRMT1 is observed in various types of human cancers and is associated with poor clinical prognosis [29–31]. Moreover, PRMT1 is an important regulator in many cellular pathways that are dysregulated in cancers [32–34]. However, whether the function of BRD4 is affected by PRMT1 remains almost unclear.

In this study, we demonstrate that BRD4 is asymmetrically methylated at R179/181/183 by PRMT1. PRMT1 was overexpressed in ovarian cancer tissues and promoted ovarian cancer progression, which was correlated to poor clinical outcomes. PRMT1 knockdown or pharmacological treatment abrogated BRD4 methylation-associated tumorigenesis. Moreover, we disclose that asymmetric methylation by PRMT1 was required for BRD4 phosphorylation and BRD4-induced ovarian cancer cell migration and invasion, which may be associated with transforming growth factor-β (TGF-β) signaling. Jumonji Domain Containing 6 (JMJD6) demethylated PRMT1-mediated BRD4 methylation and reversed the effects of BRD4 methylation in part. Taken together, our findings may provide a basis for the development of a potential therapeutic strategy by targeting PRMT1-mediated asymmetric arginine methylation for ovarian cancer treatment.

## Results

### PRMT1 methylates BRD4-ADMA formation on Arg179/181/183

**Fig. 1:**
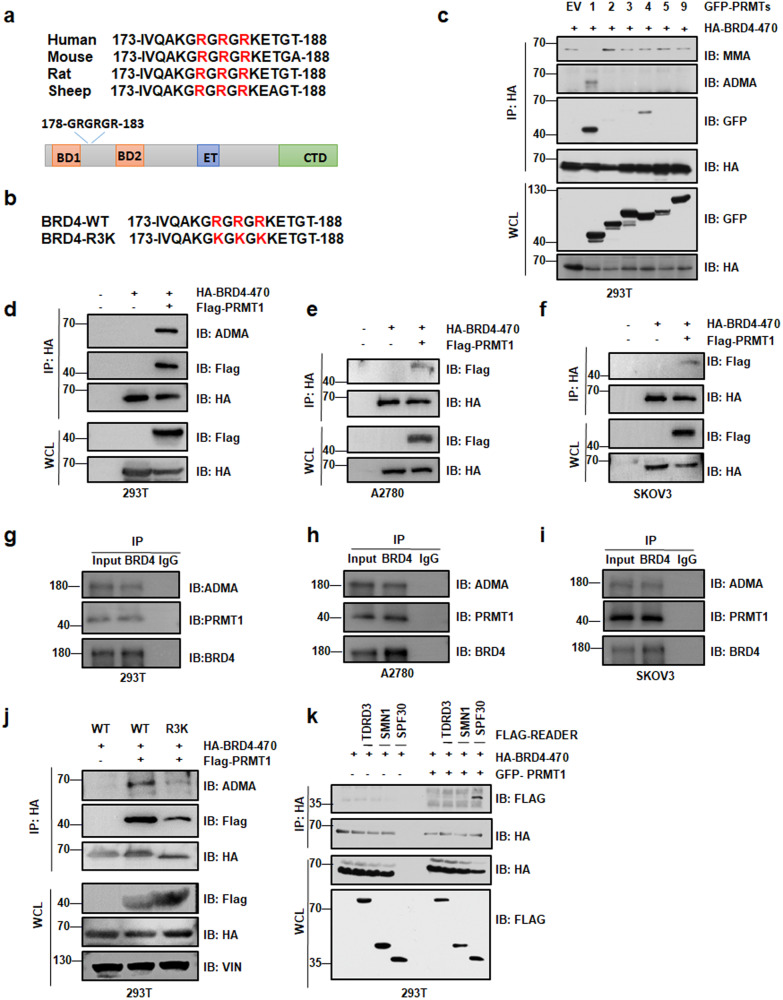
PRMT1 dimethylates BRD4 at arginine 179, 181, and 183. **a** Sequence alignments of BRD4 in mammals including humans, mice, rats, and sheep. BRD4-R179/181/183 site is denoted in the protein sequences. **b** The amino acid sequence of BRD4 wide type and BRD4 mutant type. **c** Immunoblot (IB) analysis of whole cell lysates (WCL) and immunoprecipitates (IP) derived from HEK293T cells transfected with HA-BRD4 (1–470aa) and various GFP-tagged PRMT constructs (GFP-PRMT1, GFP-PRMT2, GFP-PRMT3, GFP-PRMT4, GFP-PRMT5, GFP-PRMT9). EV empty vector. **d** IB analysis of WCL and IP analysis of ADMA formation and PRMT1 interaction of exogenous BRD4 in 293T cells transfected with HA-BRD4-470 and Flag-PRMT1. **e-f** IB analysis of WCL and IP analysis of PRMT1 interaction of exogenous BRD4 in A2780 and SKOV3cells transfected with HA-BRD4-470 and Flag-PRMT1. **g**–**i** IB analysis of WCL and IP analysis of ADMA formation and PRMT1 interaction of endogenous BRD4 in 293T cells, A2780 cells, and SKOV3 cells. **j** IB analysis of WCL and IP derived from HEK293T cells transfected with Flag-PRMT1 and HA-BRD4-WT or HA-BRD4-R3K (R179/181/183K) mutants. **k** IB analysis of WCL and IP derived from HEK293T cells transfected with HA-BRD4 (1–470aa), GFP-PRMT1 and various Flag-tagged methylation readers (Flag-TDRD3, Flag-SMN1, Flag-SPF30).

To explore whether BRD4 undergoes arginine methylation, we used a web-based database (https://www.phosphosite.org/homeAction.action) and performed mass spectrometry (MS) analysis of BRD4 protein in HEK293T cells. The results revealed that arginine residues at 179, 181, and 183 (R179, R181, and R183) were methylated (Fig. S1a, b, Supplement files 1, 2). The methylation site of R179/181/183 on BRD4 is highly conserved in mammals (Fig. 1a). To determine which PRMTs methylated BRD4, we assessed the potential interactions between PRMTs (PRMT1, PRMT2, PRMT3, PRMT4, PRMT5, and PRMT9) and BRD4 by using immunoprecipitation (IP) and coimmunoprecipitation (co-IP) in HEK293T cells. We found that PRMT1 and PRMT4 could interact with BRD4 and underwent ADMA modification (Fig. 1c), which was consistent with the recent study also showed PRMT2/4-mediated arginine methylation was pivotal for BRD4 transcription and DNA repair [35]. Further experiments in A2780, SKOV3, SiHa, and HeLa cells revealed that ADMA formation on BRD4 is mediated by PRMT1 and that both endogenous and exogenous BRD4 interact with PRMT1 (Figs. 1d–i, S1c–e). As shown in Fig. 1b, to precisely localize the methylation site on BRD4, site mutagenesis to lysine (K) was carried out on R179/181/183 (R179/181/183K or R3K). Interestingly, Co-IP analysis showed that the ADMA was sharply reduced in cells with BRD4-R3K mutation as compared to BRD4-WT (Fig. 1j), indicating that the methylation assays of substitution of BRD4-R3K mutation strongly blocked PRMT1-mediated methylation of BRD4. In addition, the Tudor domains of TDRD3, SPF30, and SMN were found to interact with various methyl-arginine proteins [36, 37]. Our results verified that only SPF30 was the Tudor domains of dimethylarginine recognition of PRMT1-mediated BRD4-ADMA (Figs. 1k, S1f). Collectively, these data support the notion that BRD4 is specifically methylated by PRMT1 at R179/181/183 residues.

### PRMT1 induces ovarian tumorigenesis and is associated with poor survival

**Fig. 2:**
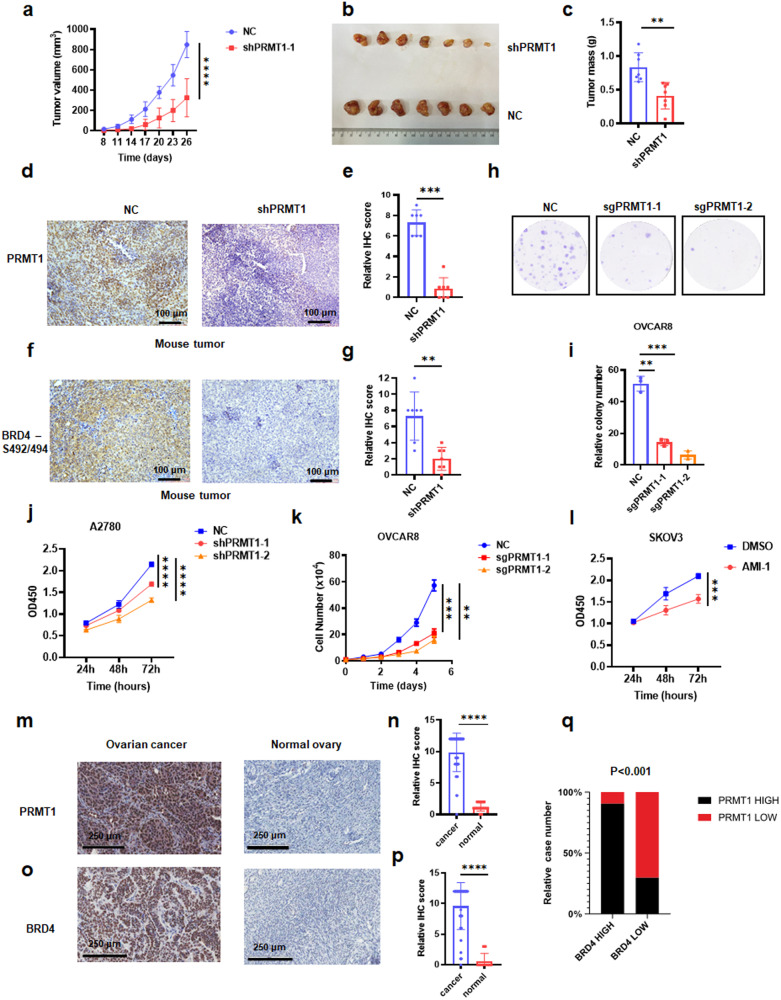
Oncogenic role of PRMT1 showed in ovarian cancer. **a**–**c** ShGFP or shPRMT1 A2780 cells were injected into the subcutaneous of nude mice (*n* = 7 mice). The in vivo tumor size was monitored (**a**). Tumors were dissected and weighed (**b** and **c**). **P < 0.01, ****P < 0.0001. **d**–**g** Representative images and statistical analysis of mouse tumor tissues derived from (**b**) stained for PRMT1 (**d** and **e**) and BRD4-S492/494 (**f**, **g**) by immunohistochemistry (IHC). Scale bar, 100 μm. **P < 0.01, ***P < 0.001. **h** and **i** Colony formation (**h**) assays were performed in PRMT1 knockout and parental OVCAR8 cells. Statistical analysis of relative colony numbers was plotted in (**i**). **P < 0.01, ***P < 0.001. **j** CCK8 assays were performed in PRMT1 knockout and parental A2780 cells for 24, 48 and 72 h. ****P < 0.0001. **k** Cell number assays were performed in PRMT1 knockout and parental OVCAR8 cells. **P < 0.01, ***P < 0.001. **l** CCK8 assay for SKOV3 cells treated with 200 μM AMI-1 for 24, 48, and 72 h. ***P < 0.001. **m**–**q** Representative images and statistical analysis of primary ovarian cancer patient samples (*n* = 80) and normal ovarian samples (*n* = 10) stained for PRMT1 (**m** and **n**) and BRD4 (**o** and **p**) by IHC. Scale bar, 200 μm. The correlations of BRD4 with PRMT1 were plotted in (**q**). ****P < 0.0001.

To further investigate the function of PRMT1 in ovarian tumor formation, a xenograft mouse model of ovarian cancer was used. In comparison to tumors derived from ovarian cancer A2780 cells, those cells with stable knockdown of PRMT1 using the shRNA approach exhibited sharply decreased tumor growth as well as reduced tumor weight (Figs. 2a–c, S2a), implying that depletion of PRMT1 attenuated ovarian tumor growth. Notably, immunohistochemical (IHC) staining displayed reduced expression of PRMT1 in shPRMT1 mouse tumors in comparison to control mouse tumors (Fig. 2d, e). However, no significant difference in BRD4 level was found in shPRMT1 mouse tumors or control mouse tumors (Fig. S2b, c).

In vitro experiment was performed to examine the functions of PRMT1 in ovarian cancer cells. Knockdown of PRMT1 profoundly blocked the proliferative activities in ovarian cancer cells, as analyzed by colony formation assay (Figs. 2h, i and S2d, e), CCK-8 assay (Fig. 2j), cell proliferation (Figs. 2k and S2f). Similar results were observed by the CCK-8 assay when using PRMT1-specific inhibitor AMI-1 (Figs. 2l and S2g, h). To assess the clinical significance of our findings, we performed an IHC analysis of tissue microarray in ovarian cancer using anti-PRMT1 and anti-BRD4 antibodies. The results showed that PRMT1 and BRD4 are markedly overexpressed in ovarian cancer tissues compared to normal tissues (Fig. 2m–p). Moreover, PRMT1 expression positively correlates with BRD4 IHC signals (Fig. 2q). Additionally, we demonstrated that high levels of PRMT1 and BRD4 were remarkably correlated with poor overall survival (Fig. S2i, j). Our results suggest that PRMT1 may exert its oncogenic roles in ovarian cancer.

### PRMT1-mediated BRD4 methylation promotes BRD4 phosphorylation

**Fig. 3:**
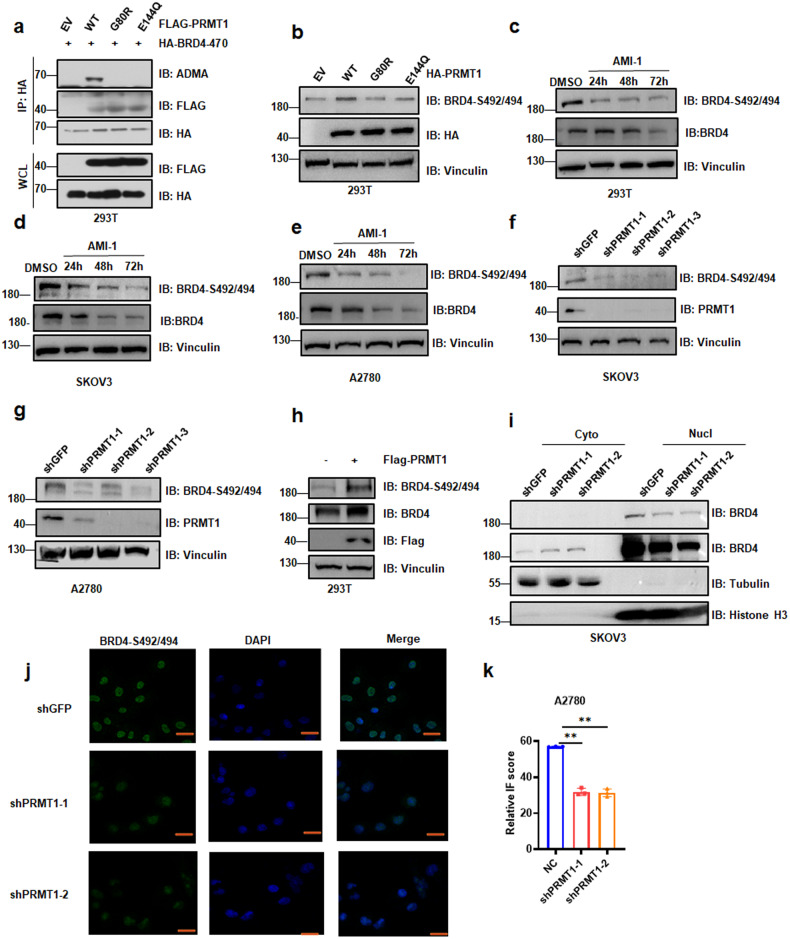
PRMT1-mediated BRD4 methylation regulates BRD4 phosphorylation. **a** IB analysis of WCL and IP derived from HEK293T cells transfected with HA-BRD4 (1–470aa), Flag-PRMT1-WT and Flag-PRMT1-mutants (Flag-PRMT1-G80R, Flag-PRMT1-E144Q). EV empty vector. **b** IB analysis of WCL derived from HEK293T cells transfected with HA-PRMT1-WT and HA-PRMT1-G80R, HA-PRMT1-E144Q. **c**–**e** IB analysis of BRD4-S492/494 derived from HEK293T cells, SKOV3 cells, and A2780 cells treated with AMI-1 in a time-dependent manner for 24, 48, and 72 h. **f** and **g** IB analysis of WCL derived from SKOV3 cells and A2780 cells transfected with shPRMT1-1, shPRMT1-2, or shPRMT1-3. **h** IB analysis of BRD4-S492/494 derived from HEK293T cells transfected with Flag-PRMT1. **i** IB analysis of BRD4 derived from cell fractionations separated from PRMT1 conditional knockout SKOV3 cells. **j** and **k** Representative images and statistical analysis of A2780 cells infected with shPRMT1 lentivirus and stained for BRD4-S492/494 by IF. Scale bar, 100 μm. **P < 0.01.

Emerging studies have demonstrated the crosstalk with arginine methylation and phosphorylation [27, 38]. Therefore, we tested whether PRMT1-mediated methylation of BRD4 may affect the phosphorylation of BRD4. Notably, PRMT1 was identified to bind with BRD4 and form ADMA modification. PRMT1-G80R and PRMT1-E144Q were catalytic inactive mutants [39, 40]. PRMT1-mediated BRD4-ADMA formation was blocked in case of PRMT1-G80R or PRMT1-E144Q (Fig. 3a). Additionally, BRD4-S492/494 protein level was enhanced by enforcing expression of Flag-PRMT1-WT, but not the mutant PRMT1 (Figs. 3b, S3a). Consistently, we identified that the expression of BRD4-phosphorylation was inhibited in a time-dependent manner when treated with the PRMT1-specific inhibitor AMI-1 in HEK293T cells, A2780 cells and SKOV3 cells (Fig. 3c–e). Similar results were also obtained in PRMT1-knockdown cells (Figs. 3f, g, S3b), while BRD4-S492/494 protein was elevated in cells with Flag-PRMT1 overexpression (Figs. 3h, S3c, d). Importantly, WB analysis showed that the knockdown of PRMT1 resulted in a lower protein level of BRD4 in the nucleus (Fig. 3i). The IF assay revealed that phosphorylation of BRD4 at Serine 492/494 was significantly attenuated in A2780-shPRMT1 cells than that in A2780-shCtrl cells (Fig. 3j, k). Moreover, IHC staining also showed a lower level of phosphorylated BRD4 in shPRMT1 mouse tumors as compared to control mouse tumors (Fig. 2f, g). Altogether, these results demonstrated that PRMT1 affects the total amount of BRD4 phosphorylated at serine 492/494.

**Fig. 4:**
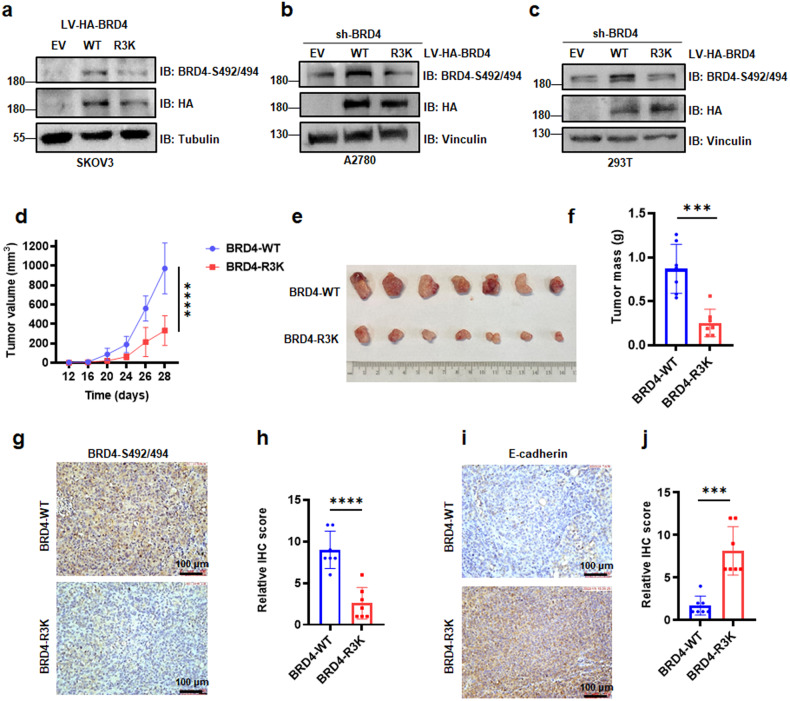
BRD4 arginine methylation at R179/181/183 affects BRD4 phosphorylation and ovarian cancer tumorigenesis. **a** IB analysis of BRD4-S492/494 derived from SKOV3 cells transfected with LV-HA-BRD4-WT and LV-HA-BRD4-R3K. **b** and **c** IB analysis of BRD4-S492/494 derived from A2780 cells and HEK293T cells in which BRD4 was knocked down to ectopically express BRD4-WT or BRD4-R3K mutant. **d**–**f** Endogenous BRD4 knocked down with shBRD4 while expressing ectopic BRD4-WT or BRD4-R3K cells were injected into the subcutaneous of nude mice (*n* = 7 mice). The in vivo tumor size was monitored (**d**). Tumors were dissected and weighed (**e** and **f**). ***P < 0.001, ****P < 0.0001. **g**–**j** Representative images and statistical analysis of mouse tumor tissues derived from (**e**) stained for BRD4-S492/494 (**g** and **h**) and E-cadherin (**i** and **j**) by IHC. Scale bar, 100 μm. ***P < 0.001, ****P < 0.0001.

Given that BRD4-R179/181/183 methylation is critical for BRD4-ADMA formation, we next construct BRD4-WT and the methylation-deficient mutant of BRD4-R3K in ovarian cancer cells. Compared to BRD4-WT, the BRD4-R3K mutation significantly inhibited the expression of BRD4-S492/494 in SKOV3 cells, as well as in A2780 cells and HEK293T cells (Figs. 4a and S3e, f). To minimize the influence of a high background of BRD4 level in A2780 cells, we first knocked down the BRD4 expression by shRNA (Fig. S3g). Then, we transfected lentiviruses expressing HA-BRD4-WT or HA-BRD4-R3K into A2780-shBRD4 cells, following which the phosphorylated BRD4 protein was remarkably upregulated in BRD4 wide type cells (Fig. 4b, c), suggesting that PRMT1-mediated BRD4 methylation promotes BRD4 phosphorylation.

### PRMT1-mediated BRD4 methylation regulates ovarian cancer cell migration and invasion

In order to determine the potential biological functions of PRMT1-mediated BRD4 methylation in vivo, we performed xenograft mouse assays with endogenous BRD4 knockdown by shBRD4 infection while expressing ectopic WT or R3K mutant BRD4 cells (A2780-shBRD4-BRD4-WT cells and A2780-shBRD4-BRD4-R3K cells). Notably, compared to the BRD4-WT group, the BRD4 mutation group significantly retarded tumor growth and tumor mass (Fig. 4d–f). Subsequently, we measured BRD4-S492/494 and E-cadherin levels in ovarian cancer tissue sections by IHC assays. The results showed that a significantly high level of BRD4-S492/494 and a low level of E-cadherin were observed in BRD4-WT tissues as compared to BRD4-R3K tissues (Fig. 4g–j).

**Fig. 5:**
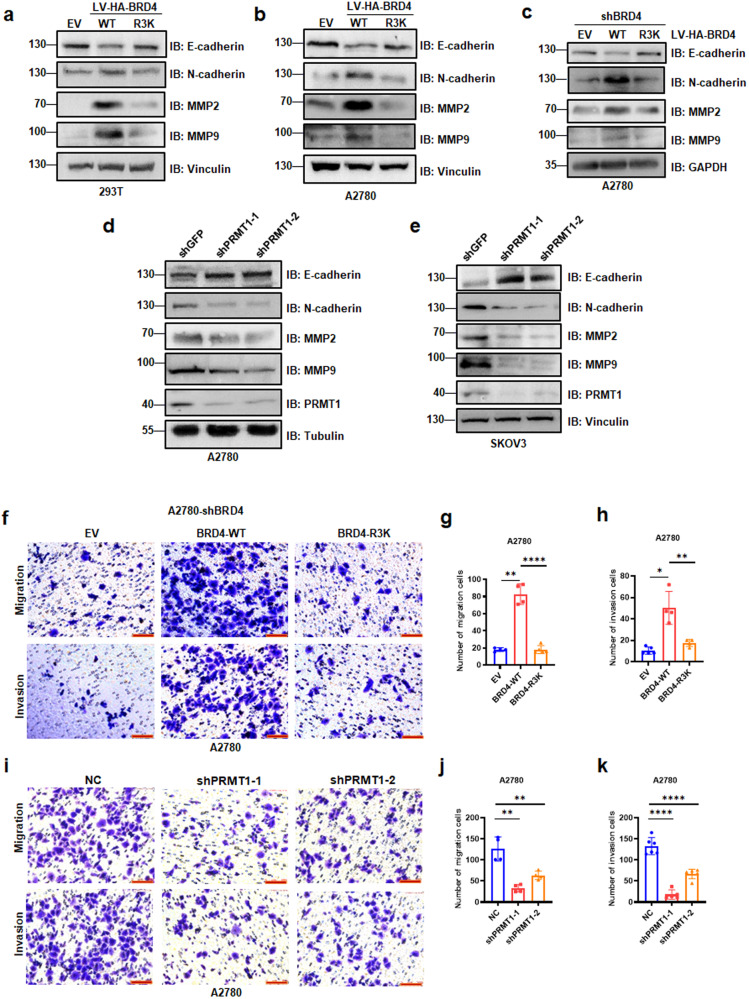
BRD4 arginine methylation promotes ovarian cancer metastasis. **a**–**c** IB analysis of E-cadherin, N-cadherin, MMP2, MMP9 derived from HEK293T cells (**a**), A2780 cells (**b**) and shBRD4-A2780 cells (**c**) infected with BRD4-WT or BRD4-R3K. **d** and **e** IB analysis of E-cadherin, N-cadherin, MMP2, MMP9 derived from A2780 cells (**d**) and SKOV3 cells (**e**) transfected with shPRMT1-1 and shPRMT1-2. **f**–**h** Representative images and statistical analysis of migration and invasion of A2780-shBRD4 cells transfected with BRD4-WT or BRD4-R3K assessed by Transwell assays. *P < 0.05, **P < 0.01, ****P < 0.0001. **i**–**k** Representative images and statistical analysis of migration and invasion of A2780 cells transfected with shPRMT1-1 or shPRMT1-2 assessed by Transwell assays. **P < 0.01, ****P < 0.0001.

We next aimed to evaluate the relationship between BRD4 methylation and ovarian cancer cell metastasis in vitro. We investigated the protein level of EMT markers such as E-cadherin, N-cadherin, MMP2, and MMP9 upon the overexpression of BRD4-WT or R3K mutant in HEK293T, A2780, and SKOV3 cells, as well as in HEK293T-shBRD4 and A2780-shBRD4 cells. E-cadherin was strongly reduced in HEK293T, A2780, SKOV3, HEK293T-shBRD4 and A2780-shBRD4 cells stably expressing BRD4-WT as compared to that in mutant BRD4 cells or vector cells, while N-cadherin, MMP2, and MMP9 were highly expressed in cells stably expressing WT-BRD4 (Figs. 5a–c and S4a, b). Furthermore, in A2780 cells and SKOV3 cells with PRMT1 depletion, we observed elevated expression of E-cadherin and decreased levels of N-cadherin, MMP2, and MMP9 in shPRMT1 cells (Fig. 5d, e). Noticeably, ovarian cancer cells with forced BRD4-WT expression showed significantly increased cell migration and Transwell invasion compared with vector cells and BRD4-mutant cells (Fig. 5f–h and S4c–h). Notably, ovarian cancer cells transfected with shPRMT1 also showed decreased cell migration and invasion (Fig. 5i–k and S4i–k). Our results suggest that PRMT1-mediated BRD4 methylation is critical for BRD4-induced EMT and metastasis.

### JMJD6 demethylates BRD4 arginine methylation

**Fig. 6:**
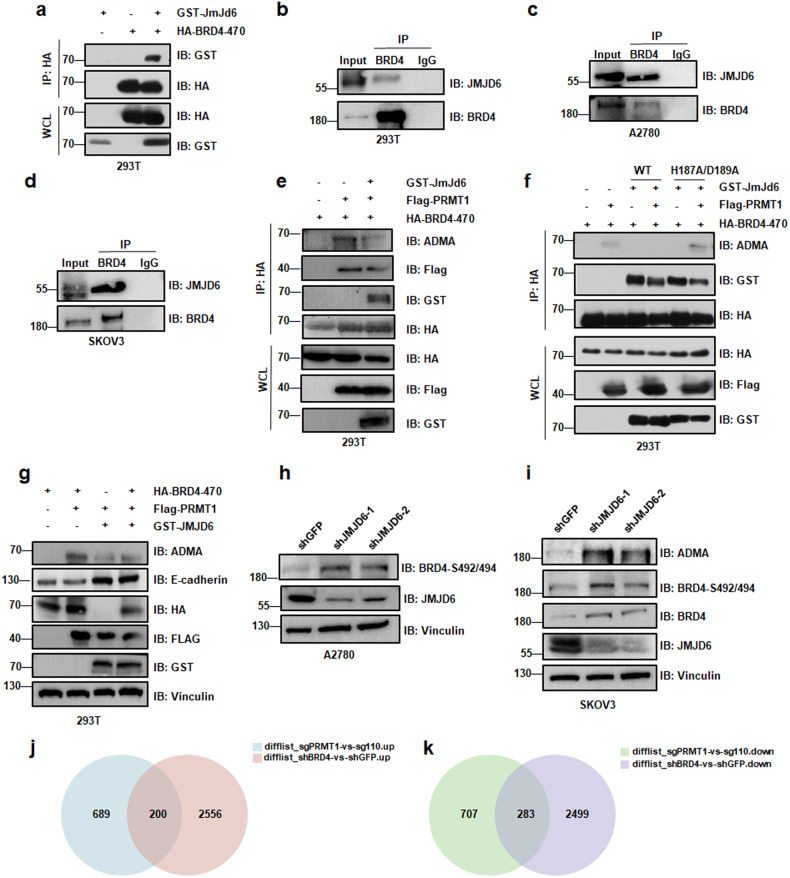
JMJD6 demethylates BRD4 to inhibit BRD4 phosphorylation. **a** IB analysis of WCL and IP of JMJD6 interaction of exogenous BRD4 in 293T cells transfected with HA-BRD4-470 and GST-JMJD6. **b**–**d** IB analysis of WCL and IP analysis of JMJD6 interaction of endogenous BRD4 in 293T cells, A2780 cells, and SKOV3 cells. **e** IB analysis of WCL and IP derived from HEK293T cells transfected with HA-BRD4-470, Flag-PRMT1, and GST-JMJD6. **f** IB analysis of WCL and IP derived from HEK293T cells transfected with HA-BRD4-470, Flag-PRMT1, GST-JMJD6-WT, or GST-JMJD6-H187A/D189A. **g** IB analysis of ADMA and E-cadherin derived from HEK293T cells transfected with HA-BRD4-470, Flag-PRMT1, and GST-JMJD6. **h** and **i** IB analysis of BRD4-S492/494 derived from A2780 cells and SKOV3 cells infected with shJMJD6. **j** and **k** The interaction of up-regulated genes in sgPRMT1 and shBRD4 (**j**). The interaction of down-regulated genes in sgPRMT1 and shBRD4 (**k**).

JMJD6, one of the Jumonji family proteins, is a histone arginine demethylase [41, 42]. Studies have indicated that JMJD6 functions as a specific eraser demethylating PRMT1 targets [43–45]. It is already known that BRD4 binds to JMJD6 through its ET domain [46]. In this manuscript, we assessed whether JMJD6 could demethylate BRD4 arginine methylation. The IP and co-IP analysis showed that JMJD6 binds with BRD4 both endogenous and exogeneous (Fig. 6a–d). Additionally, demethylation assays indicated that the wild type of JMJD6 reduced the methylation of BRD4-ADMA in the presence of PRMT1 (Fig. 6e, g). Interestingly, JMJD6-WT, but not the catalytically inactive JMJD6-H187A/D189A [45] efficiently attenuated the methylation of BRD4 at R179/181/183 (Fig. 6f). JMJD6-depletion concomitantly elevated BRD4-S492/494 expression in various ovarian cancer cells (Fig. 6h, i). Hence, these results suggest that JMJD6 antagonizes PRMT1-mediated BRD4 arginine methylation.

### PRMT1-mediated BRD4 methylation is correlated with TGF-β

**Fig. 7:**
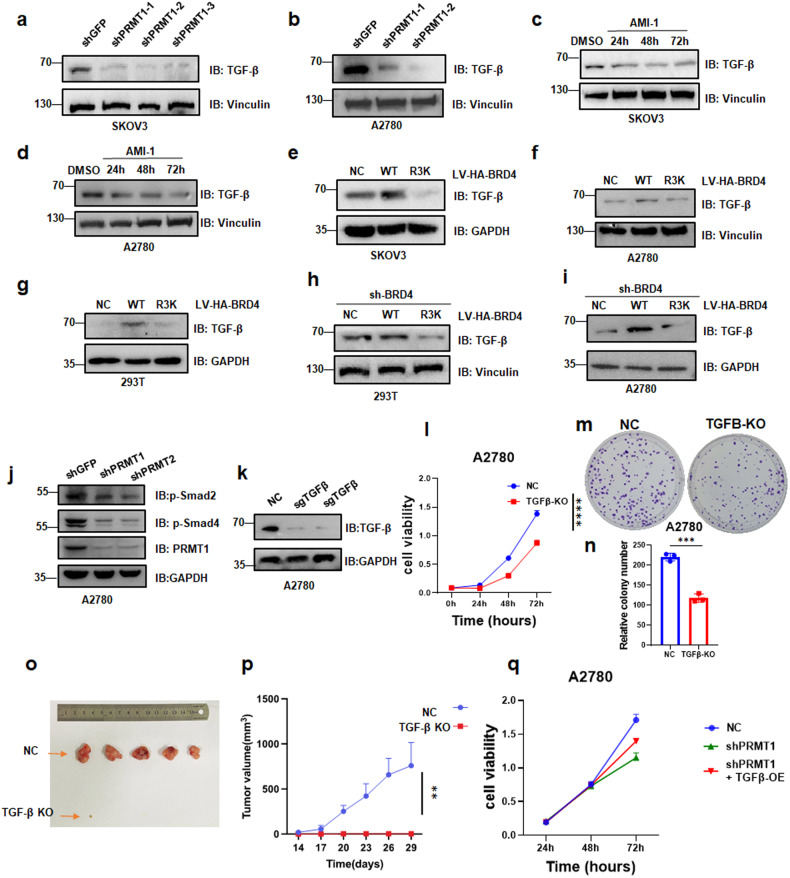
PRMT1-mediated BRD4 methylation is correlated with TGF-β. **a** and **b** IB analysis of TGF-β derived from SKOV3 cells and A2780 cells transfected with shPRMT1. **c** and **d** IB analysis of TGF-β derived from SKOV3 cells and A2780 cells treated with AMI-1 for 24, 48, and 72 h. **e**–**g** IB analysis of TGF-β derived from SKOV3 cells, A2780 cells, 293T cells stably expressed HA-BRD4-WT and HA-BRD4-R3K mutant. **h** and **i** IB analysis of TGF-β derived from BRD4 knockdown 293T cells and BRD4 knockdown A2780 cells stably expressed HA-BRD4-WT and HA-BRD4-R3K mutant. **j** IB analysis of p-Smad2 and p-Smad4 derived from A2780 cells transfected with shPRMT1-1 and shPRMT1-2. **k** IB analysis of TGF-β derived from A2780 cells infected with sgTGF-β. **l** CCK8 assays were performed in TGF-β knockout A2780 ovarian cancer cells for 24, 48, and 72 h. Statistical analysis of relative colony numbers was plotted. ****P < 0.0001. **m** and **n** Colony formation were performed in TGF-β knockout A2780 cells. Statistical analysis of relative colony numbers was plotted. ***P < 0.001. **o** and **p** A2780-WT or TGF-β knockout (TGF-β KO) cells were injected into the subcutaneous of nude mice (*n* = 5 mice). The in vivo tumor size was monitored and tumors were dissected. ** *P* < 0.01. **q** CCK8 assays were performed in A2780 cells transfected with sh-PRMT1 and cells transfected with shPRMT1 and TGF-β for 24, 48, and 72 h.

We hypothesized that PRMT1-mediated BRD4 methylation could serve as a regulatory mechanism to inference downstream targets and pathways. To address this hypothesis, we designed an RNA-seq experiment using OVCAR8 control (sg110) and CRISPR KO sgPRMT1 cells and OVCAR8 control (shGFP) and BRD4 knockdown shBRD4 cells. Total RNAs were extracted from these cells, and triplicate samples were sent for sequencing (Supplement file 3). We found 889 upregulated and 990 downregulated genes in the PRMT1 knockout cells, and identified 2756 upregulated and 2782 downregulated genes in the BRD4 knockdown cells (Fig. S5a, b, heatmap, Fig. S5c). Then, in order to further explore the link between PRMT1 and BRD4, we compared the up- and down-regulated genes in the sgPRMT1 cells to shBRD4 cells and found 200 genes were upregulated, 283 genes were downregulated both in sgPRMT1 cells and shBRD4 cells (Fig. 6j, k). Kyoto Encyclopedia of Genes and Genomes (KEGG) pathways were carried out to discover diverse pathways in the 200 upregulated genes and 283 downregulated genes (Supplement file 4). Among them, we noticed that the TGF-β signaling pathway may associated with PRMT1 and BRD4 (Fig. S5d, e). We hypothesized that PRMT1-mediated BRD4 modification could be associated with TGF-β. To address this hypothesis, an RT-qPCR assay was used to validate the mRNA level of TGF-β associated genes ID1, ID2, FST, and INHBE. The results showed that the mRNA level of ID1 and ID2 was higher in PRMT1 knockdown cells, while FST and INHBE were lower expressed in PRMT1 knockdown cells as compared to the control cells (Fig. S5f–i). The expression of TGF-β was then validated by western blot analysis in PRMT1-depleted cells and in BRD4-R3K mutant cells. The results showed that the expression of TGF-β was significantly decreased in SKOV3 and A2780 cells with PRMT1 knockdown (Fig. 7a, b). Similar results were obtained in ovarian cancer cells treated with AMI-1 (Fig. 7c, d). However, Knockdown of TGF-β did not affect PRMT1 (Fig. S3h). Furthermore, the protein level of TGF-β was also highly expressed in cells stably expressed BRD4-WT as compared to control cells and stably expressed BRD4-R3K mutant cells (Fig. 7e–i). Notably, the expression of phospho-Smad2 and phospho-Smad4 was significantly decreased in A2780 cells after PRMT1 knockdown (Fig. 7j). Then, in order to test the cell function of TGF-β in ovarian tumor, TGF-β knockout A2780 cells were established (Fig. 7k). CCK-8 assay and colony formation assay for TGF-β knockout A2780 cells showed that inhibition of TGF-β profoundly blocked the proliferative activities in ovarian cancer cells (Fig. 7l–n). And in vivo experiment of a xenograft mouse model by injecting TGF-β knockout A2780 cells into mouse subcutaneously demonstrated that in comparison to the control group, the TGF-β knockout group exhibited sharp inhibition in ovarian tumor growth, which showed that only one mouse developed a very small tumor in TGF-β knockout group (Fig. 7o, p), implying that depletion of PRMT1 attenuated ovarian tumor growth. Furthermore, the cell compensation test showed that TGF-β could counteract the inhibition of cell growth induced by PRMT1 knockdown (Fig. 7q). Therefore, these results together support the notion that PRMT1-mediated BRD4 methylation is associated with the TGF-β pathway.

## Discussion

PTMs of BRD4 are crucial for regulating its protein stability, biological process, and function. For instance, Cullin3^SPOP^-targeted BRD4 ubiquitination promotes BRD4 protein degradation, while prostate cancer-associated SPOP mutants block this process [47]. Besides ubiquitination, phosphorylation of BRD4 N-terminal sites is crucial for the ability of BRD4 binding to chromatin and regulating specific transcription factors, such as p53 tumor suppressor protein, NF-κB and high-risk HPV-encoded E2 protein [48, 49]. It has been reported that BRD4 is methylated at lysine-99 by SETD6 to specifically determine the recruitment of the transcription factor E2F1 to target genes implicated in mRNA translation [16]. Here we reported that BRD4 was methylated by PRMT1 at R179/181/183 and formed ADMA modification, which can be reversed by JMJD6.

PRMT1 is involved in the process of carcinogenesis in amounts of tumors, such as acute myeloid leukemia, pancreatic cancer, and colorectal cancer [38, 50–52]. A recent study revealed that PRMT1-mediated NONO arginine methylation at R251 in colorectal cancer cells [53]. PRMT1 was highly expressed in the colorectal cancer tissues and exhibited oncogenic function in colorectal cancer, which was associated with poor overall survival in advanced colorectal cancer patients [53]. Similarly, another study illustrated that methylation of EZH2 by PRMT1 at R342 promoted breast cancer cell EMT, invasion, and metastasis, and high levels of EZH2 methylation was associated with poor clinical outcome in breast cancer patients [28]. PRMT1 was also reported to be involved in ovarian cancer cisplatin. PRMT1 arginine methylation controlled the response to cisplatin in ovarian cancer cells [54]. The inhibition of PRMT1 suppressed the cancer cells' growth when exposed to low doses of cisplatin, which sensitized them to apoptosis [55]. In the present work, we found that PRMT1 was overexpressed in ovarian cancer tissues, and high expression of PRMT1 was correlated to poor clinical outcomes in ovarian cancer patients. Additionally, in vivo and in vitro experiments proved that PRMT1 enhanced ovarian cancer cell proliferation and ovarian cancer tumorigenesis. Notably, PRMT1 affected ovarian cancer metastasis via an asymmetric methylation of BRD4. AMI-1, the specific inhibitor of PRMT1 [56], significantly reduced the cell proliferation of ovarian cancer and abolished the BRD4-ADMA modification in ovarian cancer. Therefore, therapeutic targeting of PRMT1-mediated arginine methylation may be a promising strategy for ovarian cancer treatment.

Emerging studies have demonstrated the crosstalk with arginine methylation and phosphorylation [27, 38]. In the present study, we reported the crosstalk between PRMT1-mediated BRD4 methylation and its phosphorylation. We found that the knockdown of PRMT1 or PRMT1 mutants or its inhibitor suppressed the expression of BRD4 phosphorylation. Similarly, BRD4 R179/181/183K mutants decreased BRD4 phosphorylation. These findings imply that there may be a positive crosstalk between PRMT1-mediated BRD4 methylation and BRD4 phosphorylation. A variety of studies have suggested that BRD4 modification is correlated to cancer progression and metastasis. BRD4 binds with Snail on acetylated lysine K146 and K187 to inhibit Snail degradation by its E3 ubiquitin ligases FBXL14 and β-TrCP1, thereby maintaining progression and metastasis in gastric cancer [57]. BRD4 phosphorylation at threonine (T) 204 induced gastric cancer cell proliferation, tumor formation, migration, and invasion, while BRD4-T204A mutation reversed this carcinogenesis [58]. Here, we described the crucial role of BRD4 arginine methylation in ovarian cancer metastasis. BRD4 methylation at R179/181/183 induced cell migration and invasion. However, in BRD4-R3K mutant cells and deficiency of PRMT1, the metastasis was inhibited in ovarian cancer cells.

One study has demonstrated that BRD4 and JMJD6 were implicated in the regulation of transcriptional pause release. JMJD6 interacted with BRD4 to erasure H4R3me2(s), and modulate Pol II promoter-proximal pause release of regulated coding genes through the activation of P-TEFb, which plays pivotal roles in developmental and transcriptional programs [59]. Our present study has also identified the interaction of JMJD6 and BRD4. Besides, JMJD6 inhibited PRMT1-mediated BRD4 methylation, which diminished BRD4-R179/181/183 modification. Knockdown of JMJD6 suppressed the expression of BRD4 phosphorylation. Regulation of the switch between methylation and demethylation of BRD4 is an essential way to control the function of BRD4 in disease progression. Although TGF-β signaling plays a vital role in the progression of tumorigenesis, the link between BRD4 and TGF-β in cancer remains mysterious. Song et al. discovered that BRD4 functional inhibitor JQ1 and silencing of BRD4 with shRNA attenuated TGF-β-induced endothelial cell migration and EMT in cardiac fibrosis through inhibition of Snail, Twist, and Slug [60]. Inhibition of BRD4 attenuated renal fibrosis by blocking TGF-β-mediated Nox4 expression [61]. Another study showed that suppression of BRD4 by JQ1 robustly blocked TGFBR2 level in pancreatic cancer cells [62]. Here we revealed that TGF-β played an oncogenic role in ovarian cancer tumorigenesis. PRMT1-mediated BRD4-ADMA modification is associated with TGF-β expression. Knockdown of PRMT1 or BRD4 mutant blocked the expression of TGF-β.

In summary, we demonstrated that PRMT1-mediated asymmetric methylation of BRD4 methylation at R179/181/183 promotes BRD4 phosphorylation and induces ovarian cancer migration and invasion. These results imply that small molecule inhibitors by targeting PRMT1-mediated asymmetric methylation might have clinical potential for the treatment of ovarian cancer.

## Materials and methods

### Cell culture

HEK293T, A2780, SKOV3, OVCAR5, OVCAR8, HeLa, and SiHa cells were obtained from the American Type Culture Collection. HEK293T, A2780, HeLa, SiHa, OVCAR5, and OVCAR8 cells were cultured in DMEM medium (Gibco) supplemented with 1% penicillin/streptomycin and 10% FBS (Gibco). SKOV3 cells were cultured in RPMI-1640 medium (Gibco) with 10% FBS. All cell lines were cultured in a 37 °C incubator with 5% CO_2_.

### Lentivirus and transfection

Cell transfection was performed using Lipofectamine (Meilunbio, MA0672) and Plus reagents following the manufacturer’s instructions. Lentiviruses were produced by co-transfecting HEK293T cells with pMD2G and psPAX2. Lentiviral Lv157-HA-BRD4, lentiviral Lv157-HA-BRD4-R179/181/183K (R3K), pcDNA3.1-HA-BRD4-R3K, pcDNA3.1-HA-PRMT1, pcDNA3.1, shGFP were purchased from Youbio Company (Shannxi, China). pcDNA3.1-GFP-PRMT1, GFP-PRMT2, GFP-PRMT3, GFP-PRMT4, GFP-PRMT5, GFP-PRMT9, Flag-PRMT1, HA-BRD4(1–470aa), HA-BRD4-R3K(1–470aa), Flag-TDRD3, Flag-SMN1, Flag-SPF30, HA-PRMT1-G80R, HA-PRMT1-E144Q, GST-JMJD6, GST-JMJD6-H187A/D189A, CRISPR/Cas9 sgJMJD6, sgPRMT1-1, sgPRMT1-2, sgPRMT1-4 and sg110 were gifts from Pro. Wenyi Wei (Harvard Medical University). ShPRMT1-1 target sequence: TGG AAG CAG ACG GTG TTC T; shPRMT1-2 target sequence: CCG TTC TGC CTG CAA GTG A; shPRMT1-3 target sequence: CGC AAC TCC ATG TTT CAT A; shBRD4 target sequence: GGA CAC GGA CTC TTA ATA A; shJMJD6-1 target sequence: AGG ACG ACT GTG TCA GCA A; shJMJD6-2 target sequence: CTA TTA CCT GGT TTA ATG T were purchased from Shanghai Jiaotong University (Shanghai, China). The lentiviruses were collected and incubated with targeting cells for 48 h, supplemented with 10 μg/ml polybrene, and then selected with 2 μg/ml puromycin or 1 mg/ml neomycin to generate stable cell lines. These ectopically expressed BRD4 is dose-dependent.

### Western blotting analysis

Western blotting was performed as described previously [63]. All primary antibodies were used at a 1:1000 dilution in TBST buffer with 5% non-fat milk for western blot. Polyclonal anti-Flag antibody (F7425), monoclonal anti-Flag antibody (F3165), polyclonal anti-HA antibody (H6908), monoclonal anti-HA antibody (H3663), anti-phospho BRD4 (Ser492/Ser494) antibody (ABE1453) were obtained from Sigma-Aldrich Company. Anti-MMA antibody (8015), anti-ADMA antibody (13522), anti-BRD4 antibody (13440), anti-GST antibody (2625), anti-JMJD6 antibody (60602), anti-Vinculin antibody (13901), anti-MMP9 antibody (13667), anti-TGF-β antibody (3709), anti-N-cadherin antibody (13116), anti-GFP antibody (2956) were obtained from Cell Signaling Technology Company (Danvers, MA). Anti-MMP2 antibodies (ab37150) were obtained from Abcam Company (Waltham, MA). Anti-E-cadherin antibodies (20874-1-AP) were obtained from Proteintech Company (Rosemont, IL). Anti-GAPDH antibody (AC001), anti-Tubulin antibody (AC008), and anti-Histone H3 antibody (A2348) were obtained from ABclonal (Woburn, MA). Anti-HA agarose beads (620030) and anti-FLAG agarose beads (580028) were purchased from Bimake (Houston, TX). Anti-Phospho-Smad2 (AF3450), and Anti-Phospho-Smad4 (AF8316) were obtained from Affinity (MA).

### Immunoprecipitation (IP)

Cells were lysed in NP-40 buffer (P0013F, Beyotime, Shanghai) supplemented with phosphatase inhibitors cocktail (Bimake, America) and protease inhibitors cocktail (Bimake, America). The protein concentrations were measured using Nanodrop (Thermo Fisher) with Bio-Rad protein assay reagent. For immunoprecipitation analysis, the lysates (1000–3000 μg) were incubated with 50% slurry of Protein A/G sepharose beads (GE Healthcare) conjugated antibody for 3–5 h at 4 °C. After washed four times with NETN buffer (0.5% NP-40, 20 mM Tris, pH 8.0, 150 mM NaCl and 1 mM EDTA), the recovered immuno-complexes were resolved by SDS–PAGE and immunoblotted with indicated antibodies.

### Mass spectrometric analysis

HEK293T cells were transfected with HA-BRD4 (1–470aa) for 2 days. Then, the cells were lysed and followed with HA immunoprecipitation. The proteins were separated in 10% SDS–PAGE and visualized using colloidal Coomassie blue staining. The band corresponding to HA-BRD4 (1–470aa) protein was excised and performed liquid chromatography–tandem mass spectrometry analysis (LC–MS/MS) in the Shanghai Luming Biological Technology, LTD (Shanghai, China).

### Mouse xenograft study

Twenty-eight female BALB/C nude mice (4–6 weeks of age) were purchased and fed by the Animal Experiment Center of Wenzhou Medical University (Wenzhou, China). Briefly, 3 × 10^6^ A2780 cells with PRMT1 knockdown and control cells were injected into the flank of 7 female nude mice, respectively. Additionally, 3 × 10^6^ A2780 cells stably expressing BRD4 WT or BRD4 R179/181/183K mutant were injected into the flank of 7 female nude mice, respectively. Tumor size was measured twice every week based on the formula *A* × *B*^2^ × 0.5, where *A* is the longest diameter and *B* represents the shortest diameter. After around 3 weeks, mice were sacrificed, and xenografted solid tumor weights were measured. The animal studies were approved by the Institutional Animal Care and Use Committee of Wenzhou Medical University (wydw2022-0512).

### Immunohistochemistry (IHC)

IHC was performed as described previously [63]. An ovarian cancer tissue microarray (Ova11039) containing 80 cases of invasive carcinoma and 10 cases of normal ovary tissues was purchased from Avilabio (Waltham, MA). The xenograft tumors were formalin-fixed, paraffin-embedded, and sliced into 4 μm sections. Then sections were deparaffinized using xylene and rehydrated in graded ethanol. After microwaved for antigen retrieval, the slices were incubated with relevant antibodies (Supplement Table 1) overnight at 4 °C. After washing with PBS, sections were incubated with the secondary antibody for 30 min. All sections were developed using 3,3-diaminobenzidine (Sigma, USA) until desired stain intensity and counterstained with hematoxylin. The protein expression level was determined as described previously [63].

### Immunofluorescence staining (IF)

IF were performed as described previously [64]. Cells were seeded on glass coverslips in a six-well cell plate and cultured overnight. Then, cells were fixed with 4% formaldehyde for 30 min and permeabilized with 0.1% Triton/PBS for 10 min. Cells were washed with PBS three times and blocked with 2% BSA in 0.1%Tween X-100/PBS for 1 h at room temperature. Then, cells were incubated with primary antibodies (Supplement Table 1) at 4 °C overnight, washed three times in PBS, and incubated with secondary antibodies for 1 h at room temperature. After being stained with DAPI (ab104139, Abcam), the slides were imaged with fluorescent microscopy (Leica Microsystems) and calculated by ImageJ. After uploading figures with fluorescence to ImageJ, change the figure type to RGB Stack. Then regulate the threshold, and calculate the fluorescence intensity with the mean gray value.

### Cell proliferation assay

CCK8 assay and colony formation assay were performed as described previously [65]. Cell viability was measured by use of the Cell Counting Kit-8 kit (Beyotime, Shanghai, China). A2780 cells (2 × 10^3^ cells per well) and SKOV3 cells (2 × 10^3^ cells per well) with PRMT1 knockdown and negative control cells were seeded on 96-well plates in DMEM supplemented with 10% FBS. After the cells were incubated for 24, 48, and 72 h, respectively, 10 μl CCK-8 reagent was added to each well and incubated at 37 °C for 3 h. Finally, the OD values were assessed at a wavelength of 450 nm for each well using a microplate reader.

Cell viability was also measured by colony formation assay. The A2780 cells and SKOV3 cells after PRMT1 knockdown and negative control cells were seeded into six-well plates (500 cells/well) and cultured with DMEM containing 10% FBS for 10 days in 37 °C at 5% CO_2_ incubator. Then, the culture media was discarded and the cells were washed with PBS three times. Afterward, we fixed the cells with 4% paraformaldehyde for 30 min. Finally, the cell colony was stained by the use of 0.1% crystal violet for 10 min. The number of cell colonies was calculated. For inhibitor treatment, 150–200 μM AMI-1 (MCE, HY-18962) was added into the A2780 and SKOV3 cells for 24, 48, and 72 h, respectively. Then, the proliferation of cells was determined by CCK8 assay.

### Cell migration and invasion assays

Experiments for determining cell migration and invasion activities were performed as described previously [66]. The Transwell chambers (Millipore, USA) were utilized to evaluate cell migration and invasion capabilities. The 100 μl cells (1 × 10^5^–1 × 10^6^ cells/ml) were seeded in serum-free DMEM in Transwell plates (8 µm pore size) in a 24-well plate. At the bottom of a 24-well plate, 500 μl DMEM containing 10% FBS was filled, leading to the upper cells across the Transwell membrane into the lower chamber. For invasion assay, the upper compartments were pre-coated with Matrigel (BD Biosciences, CA). After 24 h incubation, the culture medium in the upper chamber was discarded and the Transwell chambers were washed with PBS two times, the chambers were fixed with 4% paraformaldehyde for 30 min before staining with 0.1% crystal violet. The migrated or invaded cells were counted under an inverted routine microscope (Nikon Instruments Inc.).

### RNA-sequence and data analysis

Total RNA was extracted from OVCAR8 cells transfected with sg110, sgPRMT1, shGFP, and shBRD4, respectively. Samples were prepared in triplicate and then processed at Beijing Genomic Institution (BGI, Shenzhen, China). The data of the KEGG pathway and Heatmap were also analyzed in BGI (https://biosys.bgi.com/#/report/login).

### Statistical analysis

Statistical analysis was performed using the GraphPad Prism 9.0 software. The significance of differences between groups was analyzed with the unpaired *t*-test with Welch’s correction. IHC analysis was compared with the rank test. The correlation between the staining of PRMT1 and BRD4 of the ovarian cancer patients was evaluated by Spearman rank correlation. Overall survival was analyzed with the Kaplan–Meier method and the log-rank test. *P* < 0.05 was considered significant. All data are shown as mean ± standard deviation (SD) of at least three independent experiments.

### Supplementary information

Supplementary figures

Supplementary Table 1

Supplementary file legends

Supplementary file 1

Supplementary file 2

Supplementary file 3

Supplementary file 4

WB original images

aj-checklist

## Data Availability

The datasets supporting the conclusions of this article are included within the article and its additional files. The unprocessed data are available from the corresponding author on reasonable request.
