# Supplementary material for: Methylation of BRD4 by PRMT1 regulates BRD4 phosphorylation and promotes ovarian cancer invasion
Source: Cell Death Dis. 2023 Sep 22;14(9):624. doi: 10.1038/s41419-023-06149-5 (PMC10517134; doi:10.1038/s41419-023-06149-5)
Supplement: Supplementary file 1 — Supplementary figures [file 41419_2023_6149_MOESM1_ESM.pdf]

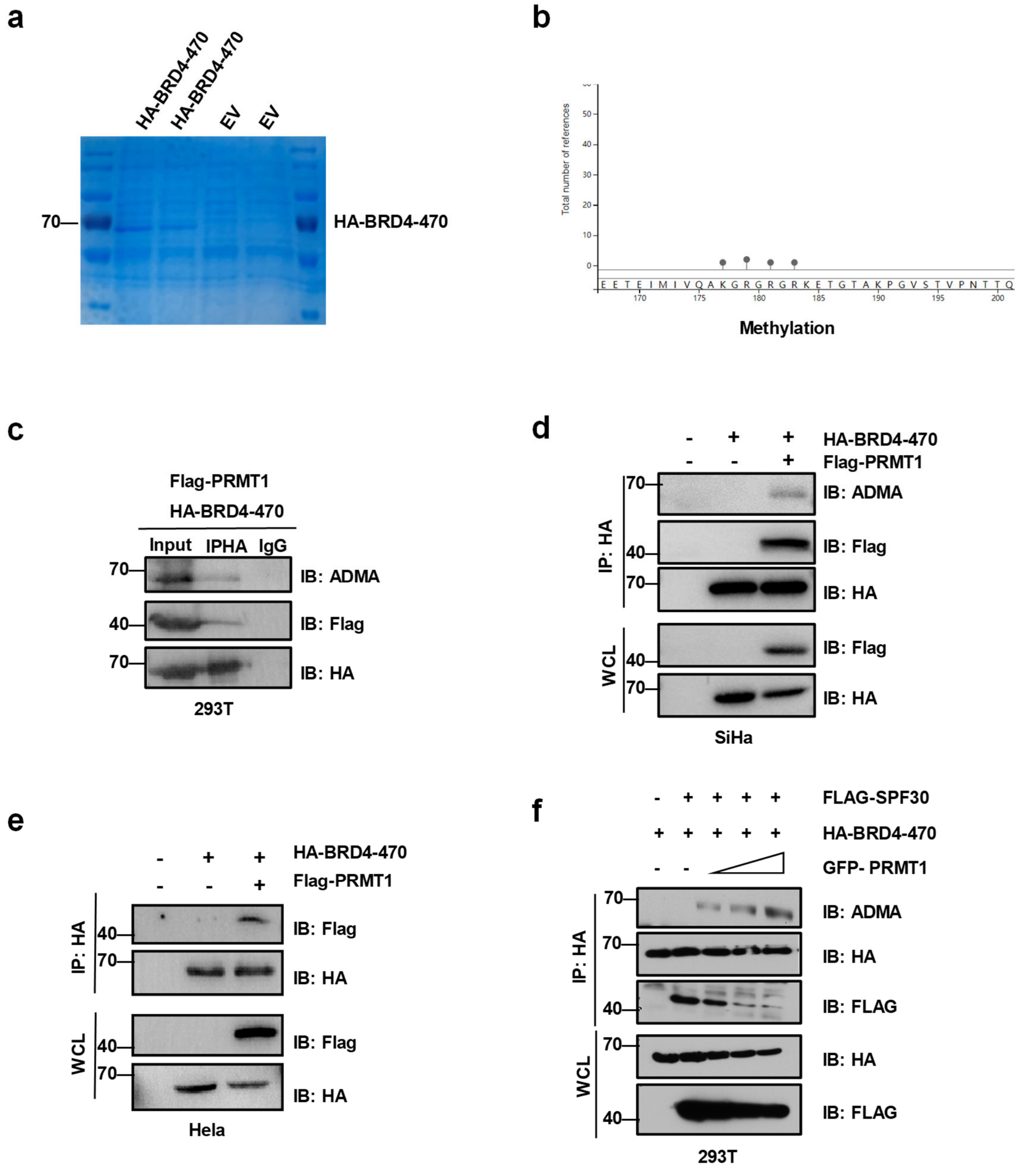

Figure S1. PRMT1 mediates BRD4 methylation.

a. The coomasie gel of BRD4 methylation. The coomasie gel of BRD4 methylation for MS analysis of methylated HA-BRD4 (1–470aa). b. The methylation of BRD4-R179/181/183 found in the website https://www.phosphosite.org/proteinAction.action?id=2335&showAllSites=true. c-e. IB analysis of WCL and IP of ADMA formation and PRMT1 interaction of exogenous BRD4. f. IB analysis of WCL and IP derived from HEK293T cells transfected with HA-BRD4 (1-470aa), Flag-SPF30, and different doses of GFP-PRMT1 (0.5-2μg).

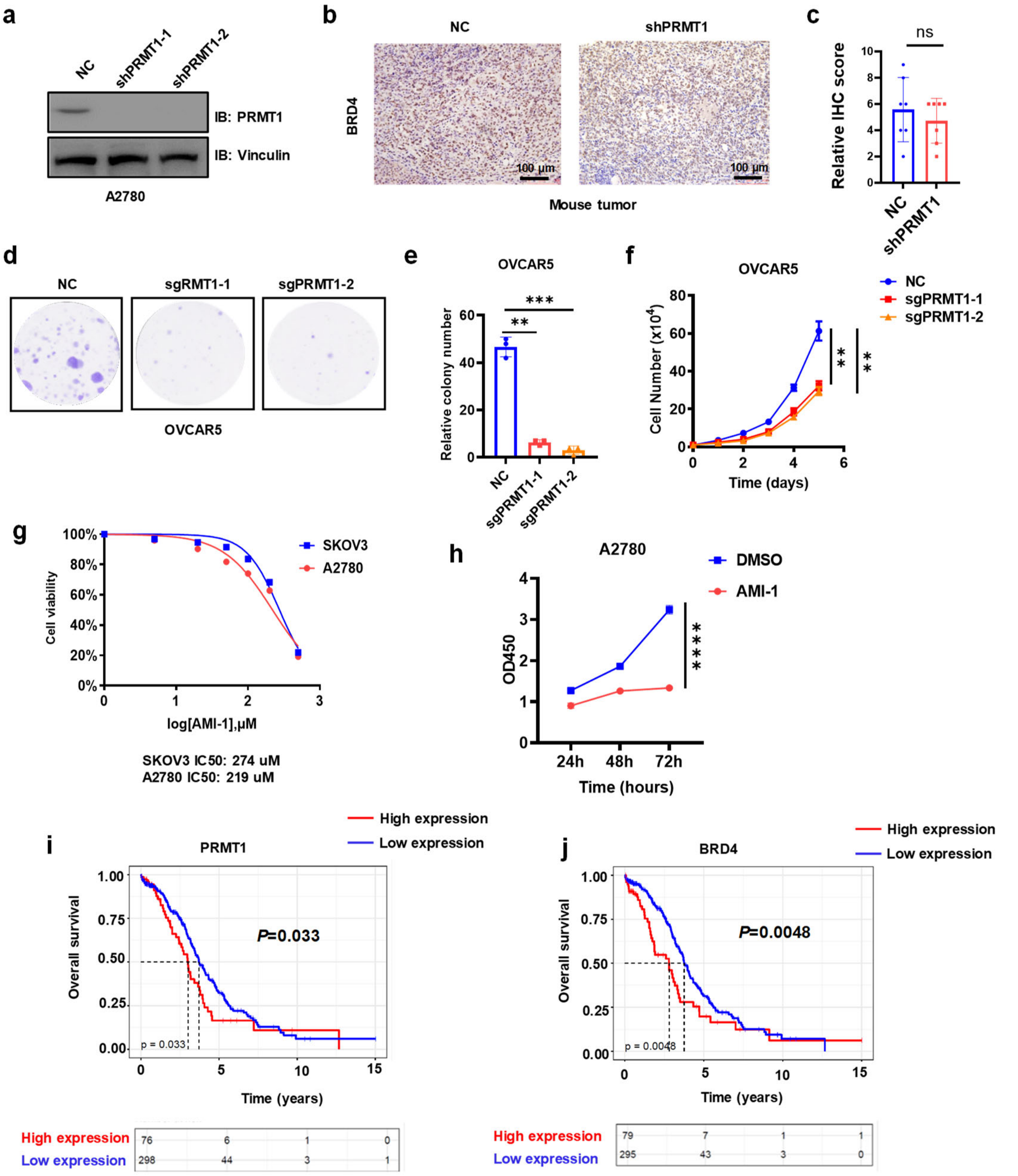

Figure S2. PRMT1 correlated with tumorigenesis and poor prognosis of ovarian cancer

a. IB analysis of WCL derived from A2780 cells infected with shPRMT1. b-c. Representative images and statistical analysis of mouse tumor tissues stained for BRD4 by IHC. Scale bar, 100 μm. ns, no significance. d-f, Colony formation (d) and relative statistic analysis (e) and proliferation (f) assays of PRMT1 knockout and parental OVCAR5 cells. ***P* <0.01, ****P* <0.001. g-h. The IC50 of AMI-1 used in A2780 cells and SKOV3 cells (g). CCK8 assay for A2780 cells treated with 150 μmol AMI-1 (h). *****P* <0.0001. i-j. Overall survival of PRMT1 and BRD4 expression in ovarian cancer patients derived from TCGA database.

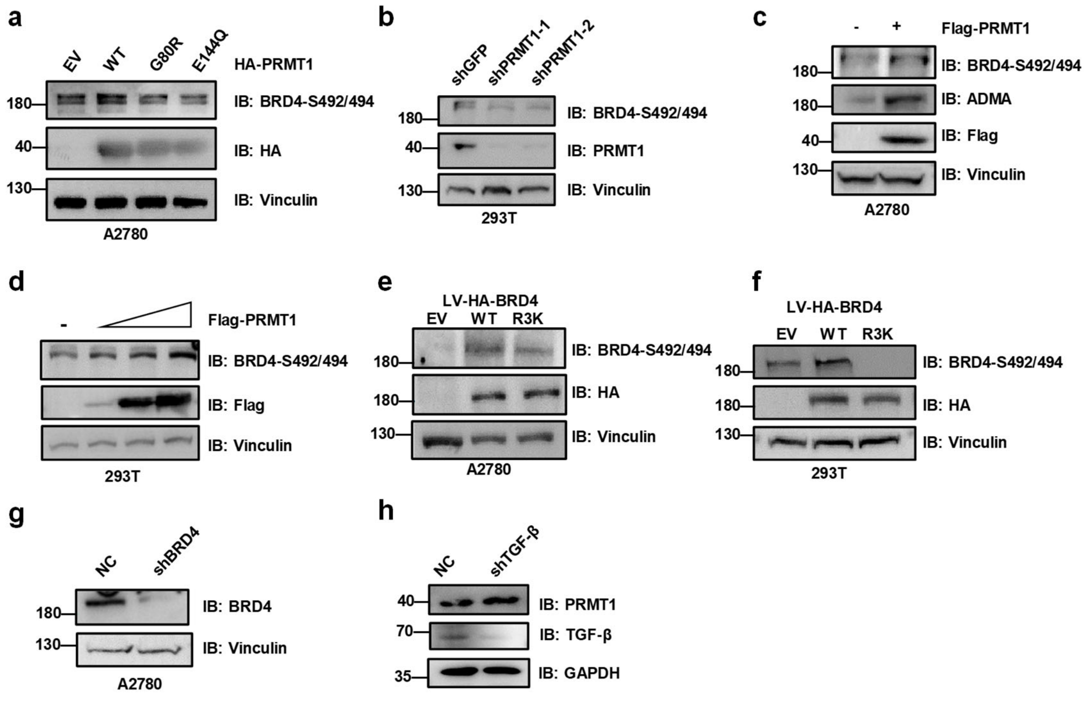

Figure S3. BRD4 methylation is correlated to the level of phosphorylated BRD4. a. IB analysis of WCL derived from A2780 cells transfected with Flag-PRMT1-WT and Flag-PRMT1-mutants. b. IB analysis of WCL derived from PRMT1 depleted HEK293T cells. c. IB analysis of WCL derived from A2780 cells transfected with Flag PRMT1. d. IB analysis of WCL derived from HEK293T cells transfected with different does of Flag-PRMT1 (0.5-2μg). e-f. IB analysis of WCL derived from A2780 and HEK293T cells stably expressed BRD4-WT or BRD4-R3K mutant. g. IB analysis of WCL derived from A2780 cells infected with shBRD4. h. IB analysis of WCL derived from A2780 cells infected with shTGF-β.

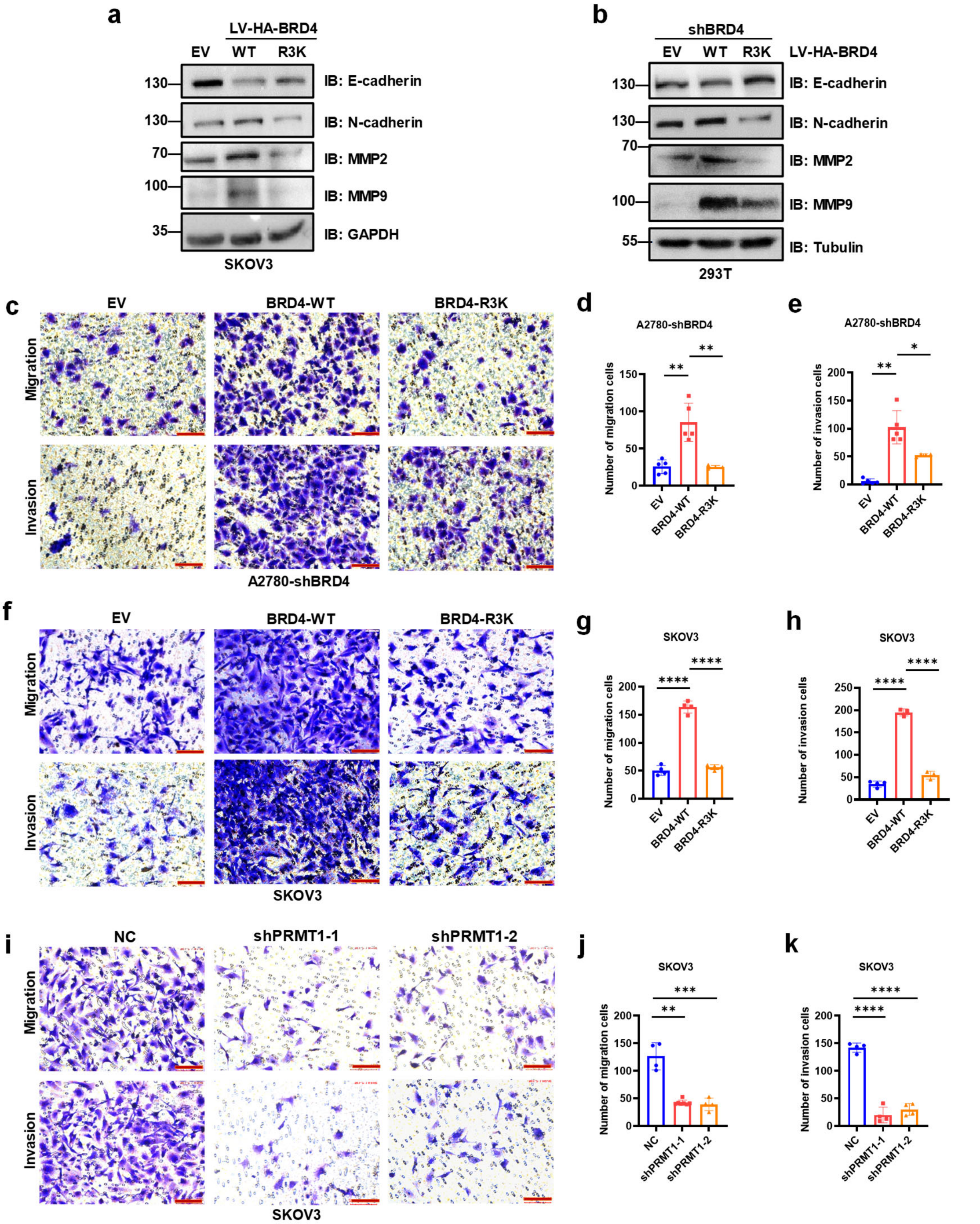

Figure S4. PRMT1-mediated BRD4 methylation is correlated to ovarian cancer migration and invasion.

a-b. IB analysis of WCL derived from indicated constructs. c-e. Representative images and statistical analysis of migration and invasion of BRD4 depleted A2780 cells transfected with BRD4-WT or BRD4-R3K assessed by Transwell assays. **P* <0.05, ***P* <0.01. f-h. Representative images and statistical analysis of migration and invasion of SKOV3 cells transfected with BRD4-WT or BRD4-R3K assessed by Transwell assays. *****P* <0.0001.i-k. Representative images and statistical analysis of migration and invasion of PRMT1 knockdown SKOV3 cells assessed by Transwell assays. ***P* <0.01, ****P* <0.001, *****P* <0.0001.

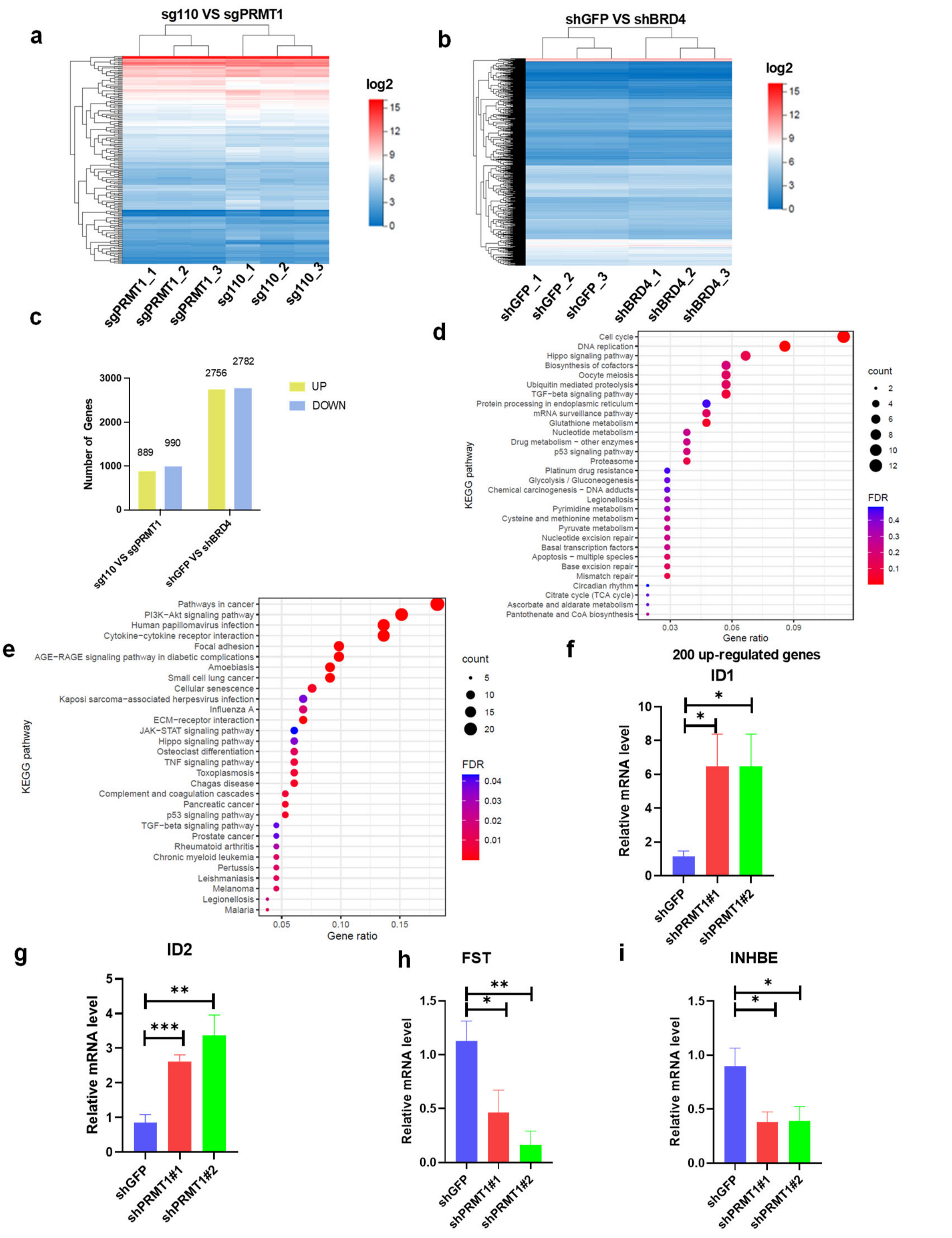

Figure S5. PRMT1-mediated BRD4 methylation is associated with TGF-β pathway.

a-b. Heatmap of differentially expressed up- and down-regulated genes from RNA-seq in OVCAR8 cells transfected with sg110 and sgPRMT1 (a), shGFP and shBRD4 (b). Rad and blue colors represent higher and lower expression, respectively. c, Differentially expressed gene in RNA sequence with indicated cells. d-e. Kyoto Encyclopedia of Genes and Genomes (KEGG) pathways analysis of 8 genes up regulated in PRMT1 knockout–dependent and BRD4 knockdown–dependent cells. f-i. RT-qPCR analysis for TGF-β associated genes.
