# Supplementary material for: Methylation of BRD4 by PRMT1 regulates BRD4 phosphorylation and promotes ovarian cancer invasion
Source: Cell Death Dis. 2023 Sep 22;14(9):624. doi: 10.1038/s41419-023-06149-5 (PMC10517134; doi:10.1038/s41419-023-06149-5)
Supplement: Supplementary file 2 — Supplementary Table 1 [file 41419_2023_6149_MOESM2_ESM.docx]

| Antibody | Source | Dilution |
| --- | --- | --- |
| anti-PRMT1 antibody | Abcam, ab190892 | 1:200 for IHC  1:500 for IF |
| anti-BRD4 antibody | Abcam, ab128874 | 1:100 for IHC |
| anti-phospho-Brd4 (Ser492/Ser494) antibody | Sigma, ABE1453 | 1:200 for IHC  1:200 for IF |
| anti-E-cadherin antibody | Proteintech, 20874-1-AP | 1:500 for IHC  1:200 for IF |

Supplement Table 1: The antibodies were used in this study.
