# Supplementary material for: Methylation of BRD4 by PRMT1 regulates BRD4 phosphorylation and promotes ovarian cancer invasion
Source: Cell Death Dis. 2023 Sep 22;14(9):624. doi: 10.1038/s41419-023-06149-5 (PMC10517134; doi:10.1038/s41419-023-06149-5)
Supplement: Supplementary file 3 — Supplementary file legends [file 41419_2023_6149_MOESM3_ESM.docx]

Supplementary file 1: Methylation-associated peptides. A web-based database (https://www.phosphosite.org/homeAction.action) regarding methylation was downloaded.

Supplementary file 2: BRD4 protein is methylated. We performed mass spectrometry (MS) analysis of BRD4 protein in HEK293T cells. The results revealed that arginine residues at 179, 181 and 183 (R179, R181and R183) were methylated.

Supplementary file 3: RNA-seq data are illustrated. We used OVCAR8 control (sg110) and CRISPR KO sgPRMT1 cells and OVCAR8 control (shGFP) and BRD4 knockdown shBRD4 cells for RNA sequencing.

Supplementary file 4: KEGG assay data. 200 genes were upregulated and 283 genes were downregulated.
