# Supplementary material for: Methylation of BRD4 by PRMT1 regulates BRD4 phosphorylation and promotes ovarian cancer invasion
Source: Cell Death Dis. 2023 Sep 22;14(9):624. doi: 10.1038/s41419-023-06149-5 (PMC10517134; doi:10.1038/s41419-023-06149-5)

Figure 1c

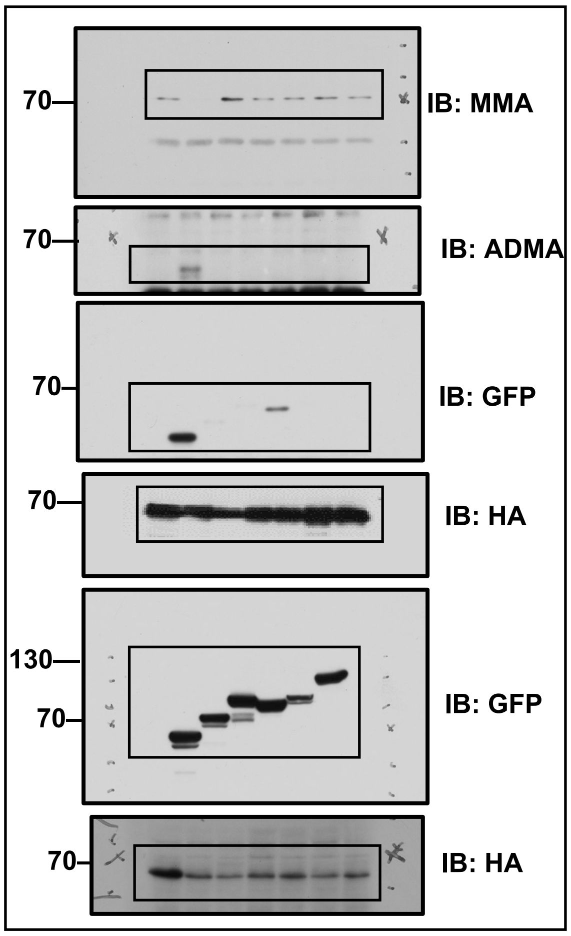

Figure 1d

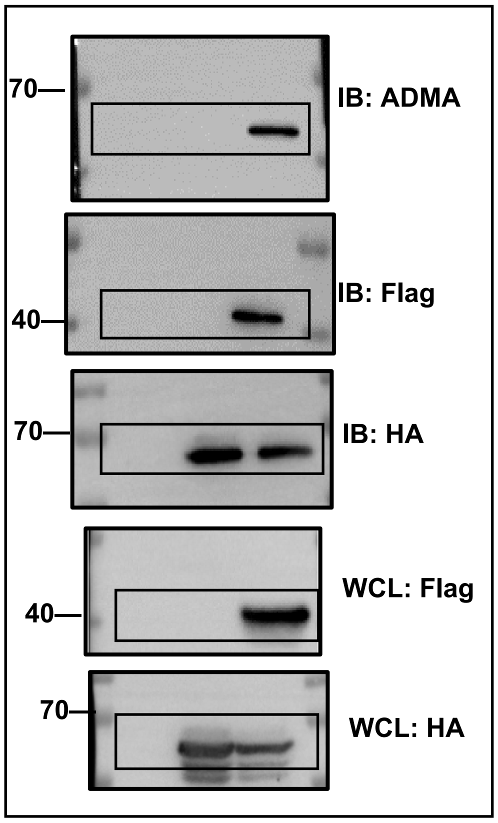

Figure 1e

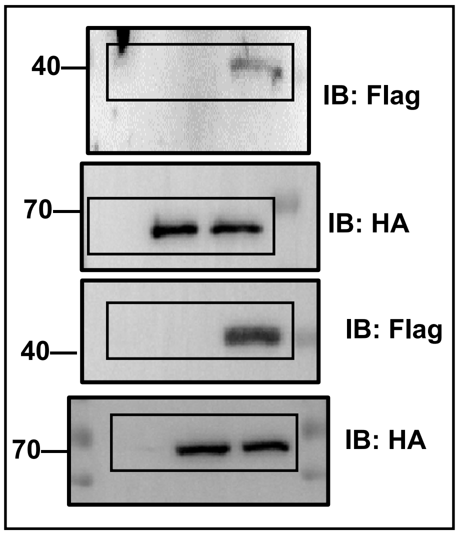

Figure 1f

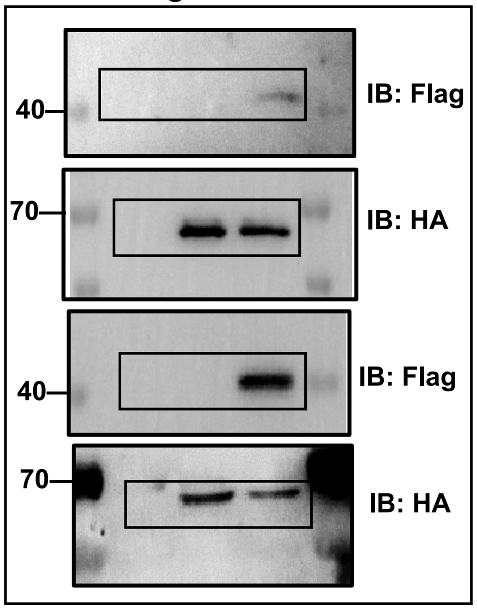

Figure 1g

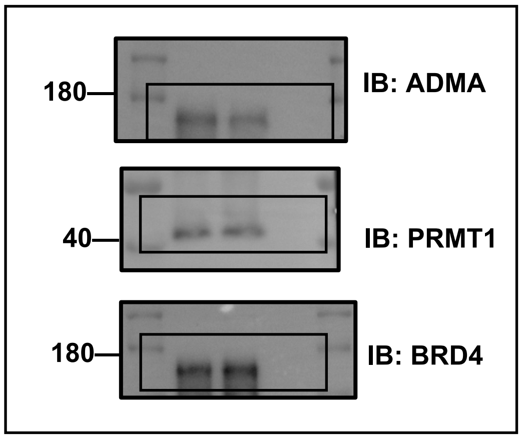

Figure 1h

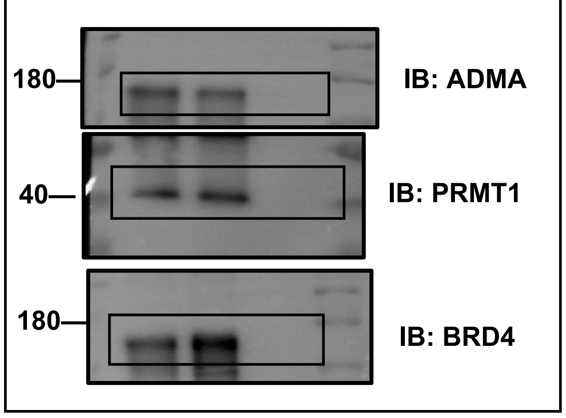

Figure 1i

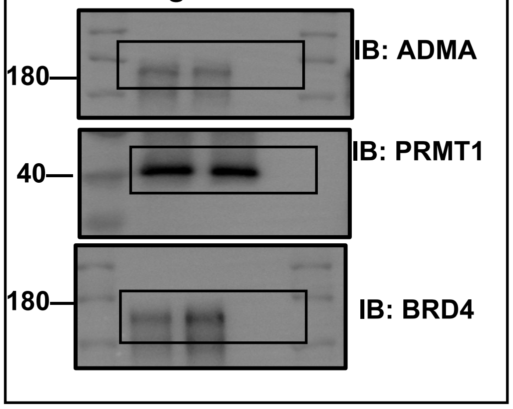

Figure 1j

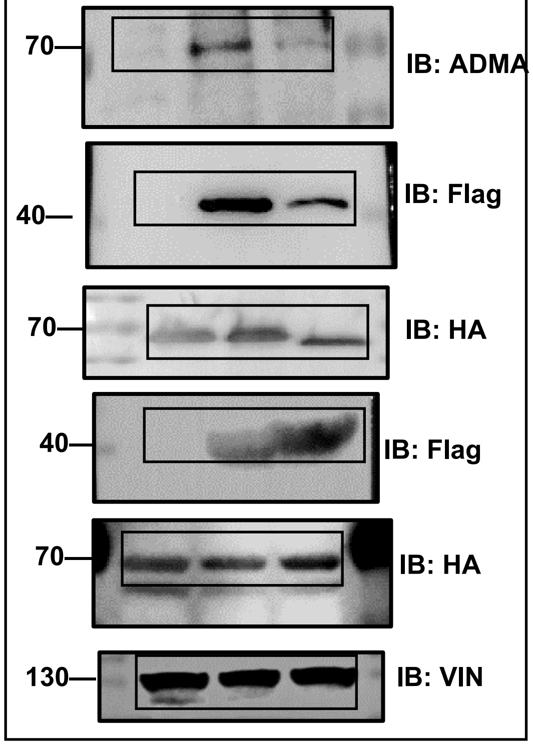

Figure 1l

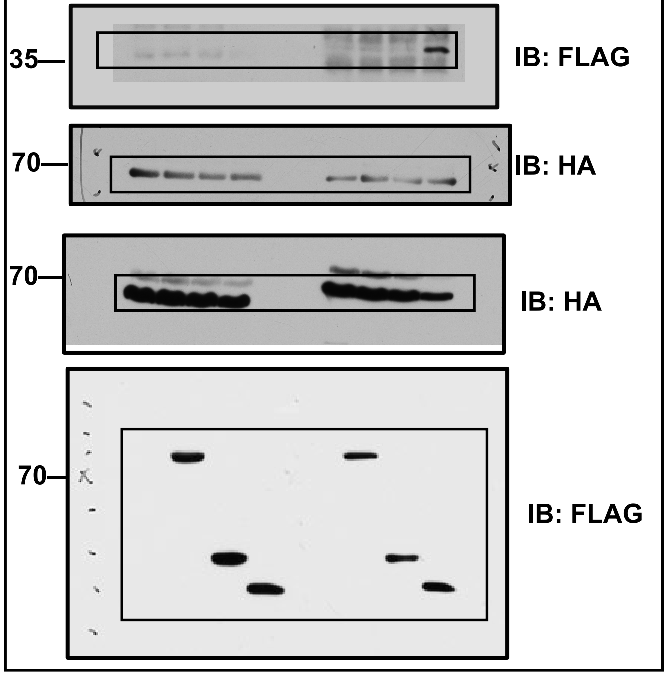

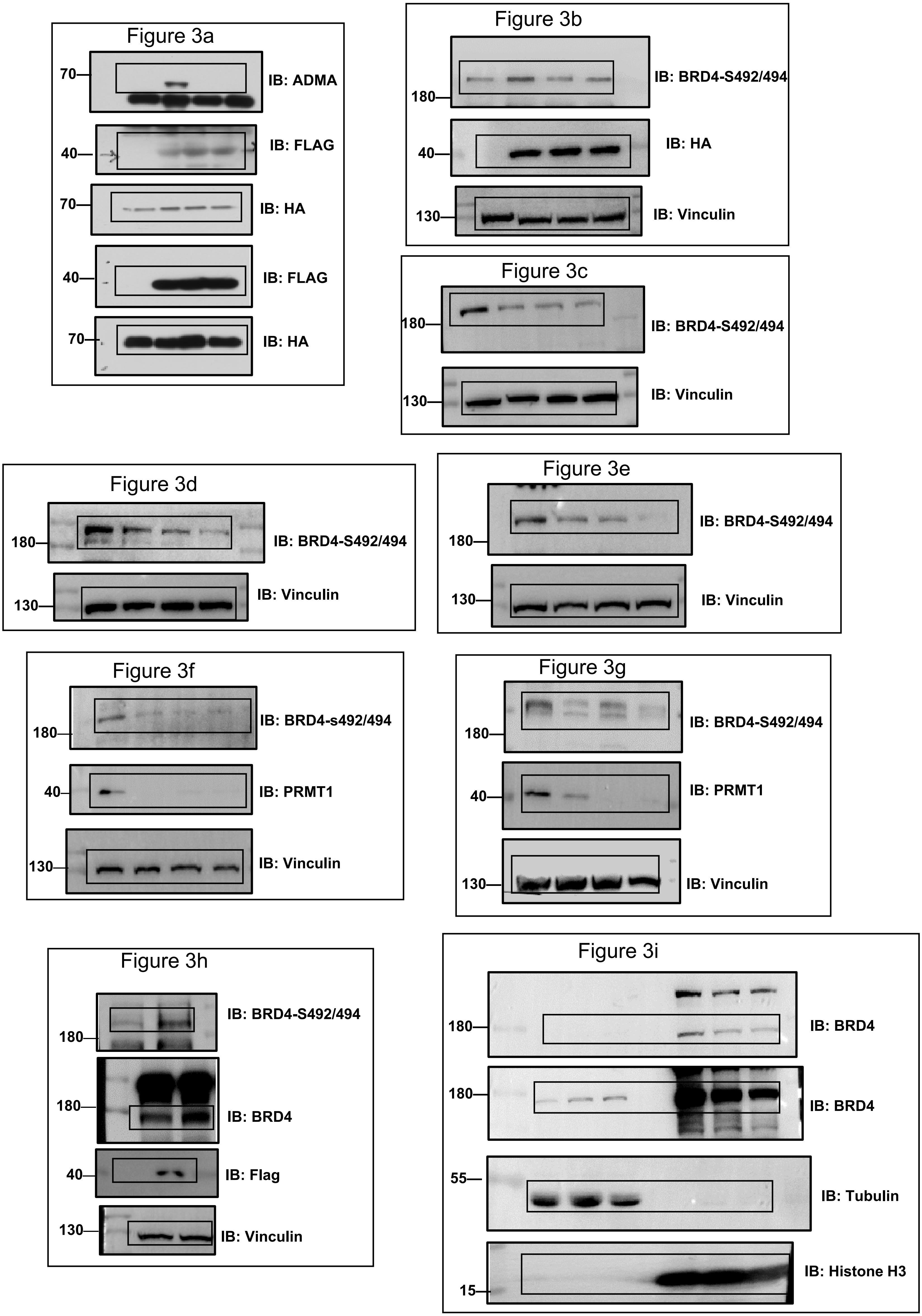

Figure 3a
70
IB: ADMA
40
IB: FLAG
70
IB: HA
40
IB: FLAG
70
IB: HA
Figure 3b
180
IB: BRD4-S492/494
40
IB: HA
130
IB: Vinculin
Figure 3c
180
IB: BRD4-S492/494
130
IB: Vinculin
Figure 3d
180
IB: BRD4-S492/494
130
IB: Vinculin
Figure 3e
180
IB: BRD4-S492/494
130
IB: Vinculin
Figure 3f
180
IB: BRD4-s492/494
40
IB: PRMT1
130
IB: Vinculin
Figure 3g
180
IB: BRD4-S492/494
40
IB: PRMT1
130
IB: Vinculin
Figure 3h
180
IB: BRD4-S492/494
180
IB: BRD4
40
IB: Flag
130
IB: Vinculin
Figure 3i
180
IB: BRD4
180
IB: BRD4
55
IB: Tubulin
15
IB: Histone H3

Figure 4a

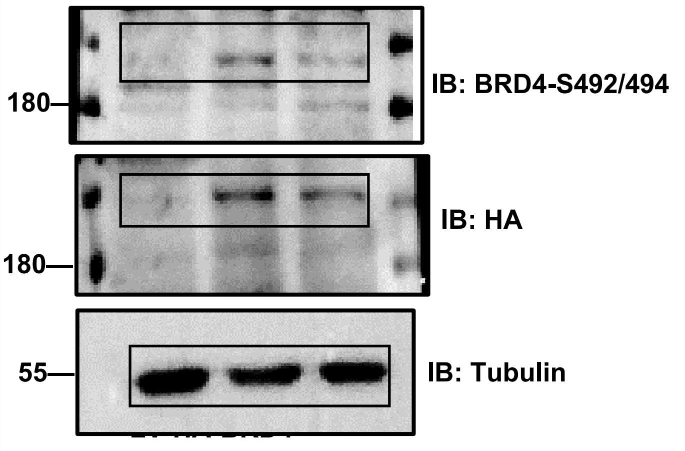

Figure 4b

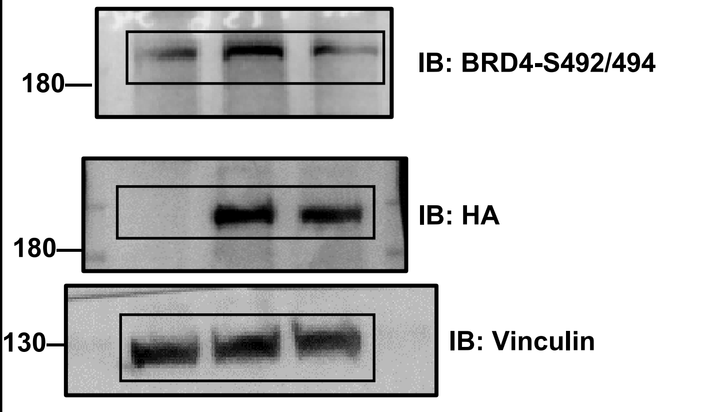

Figure 4c

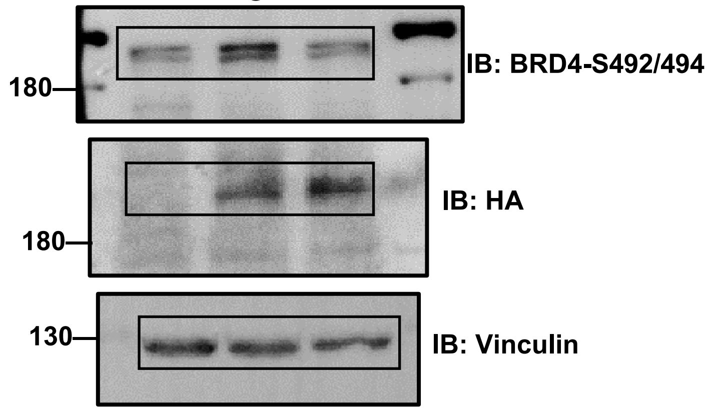

Figure 5a

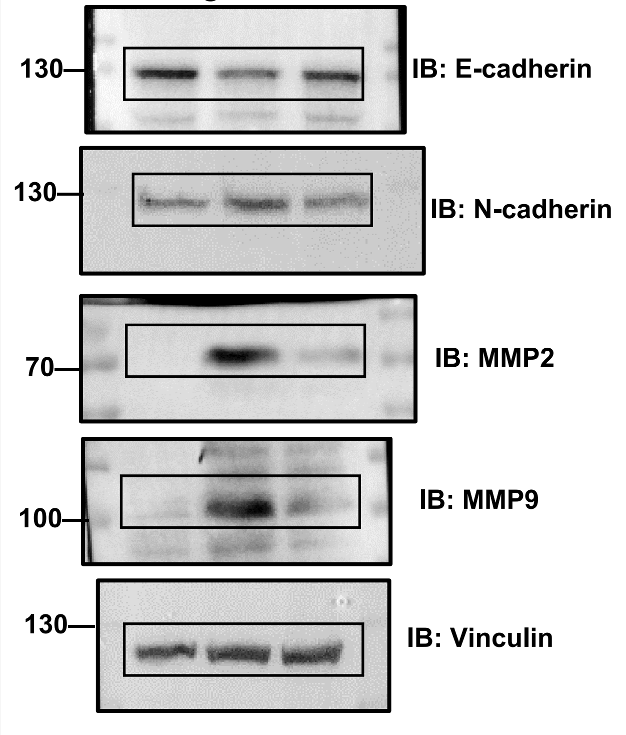

Figure 5b

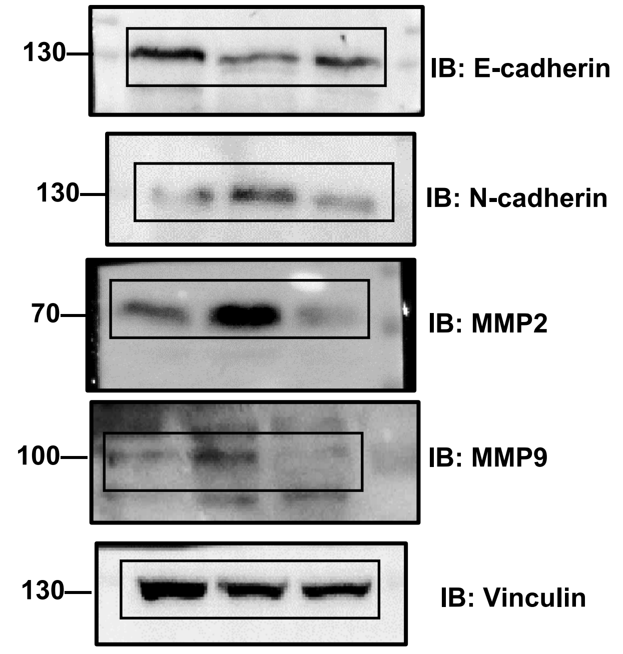

Figure 5c

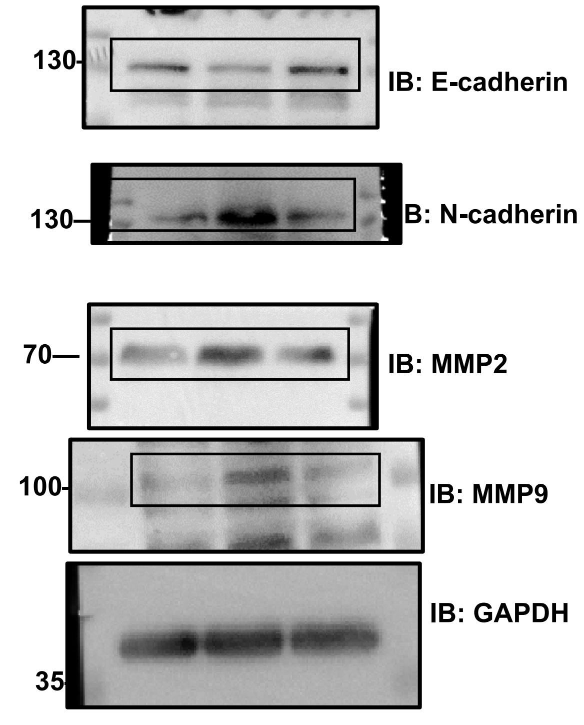

Figure 5d

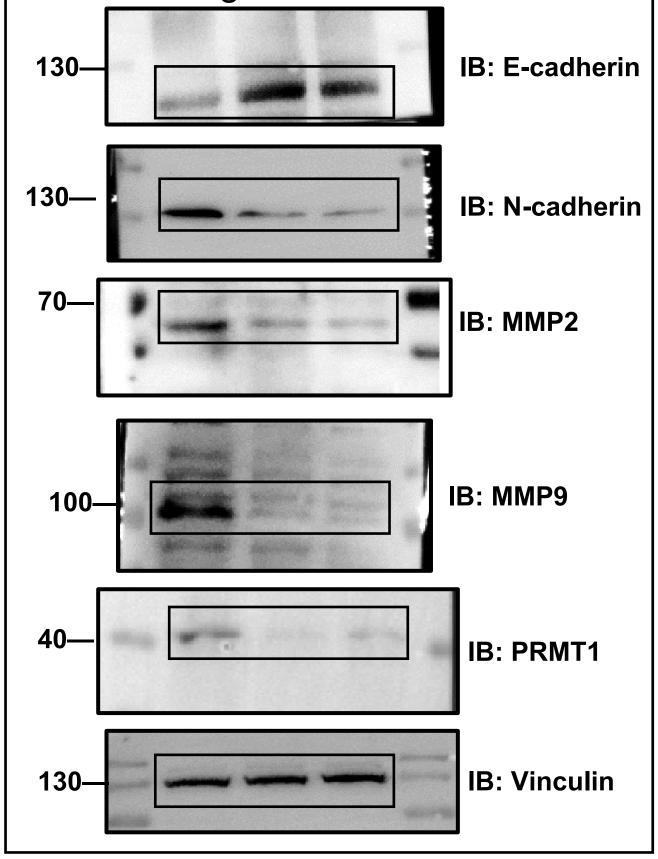

Figure 5e

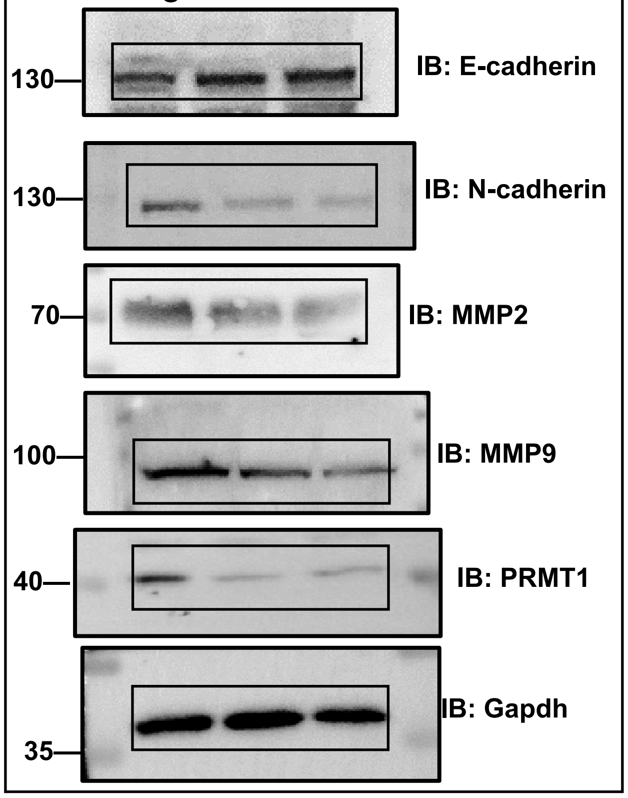

Figure 6a

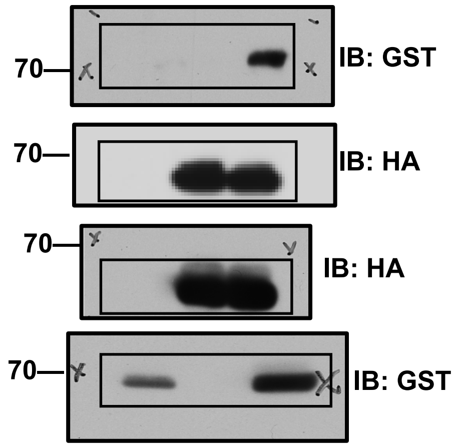

Figure 6b

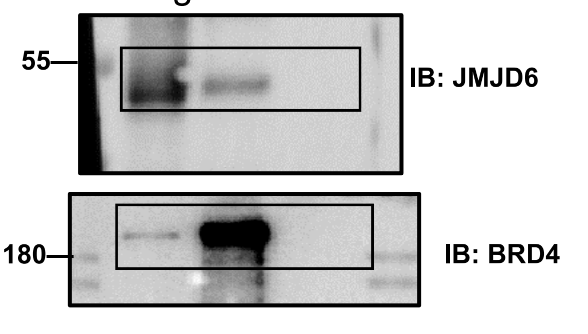

Figure 6c

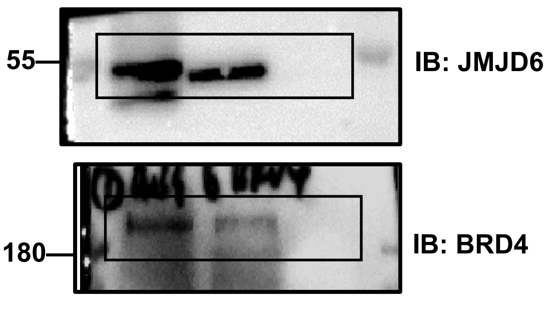

Figure 6d

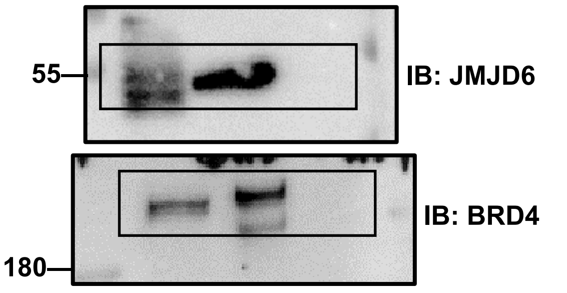

Figure 6e

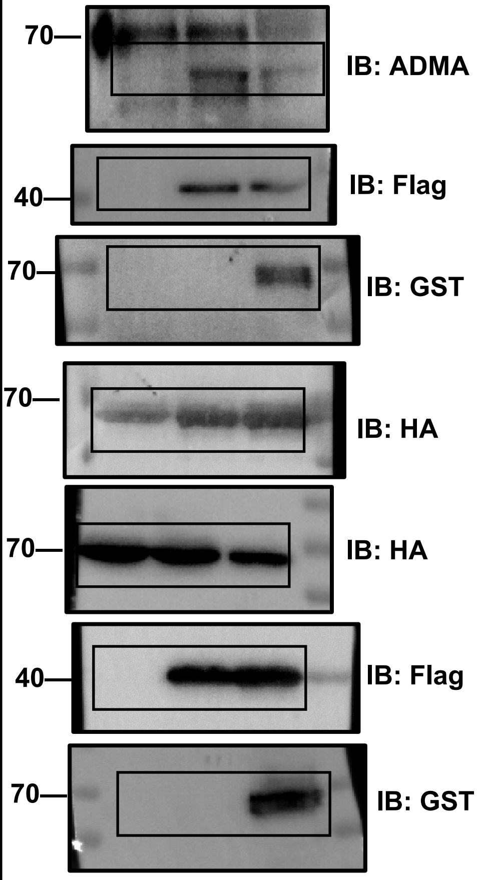

Figure 6f

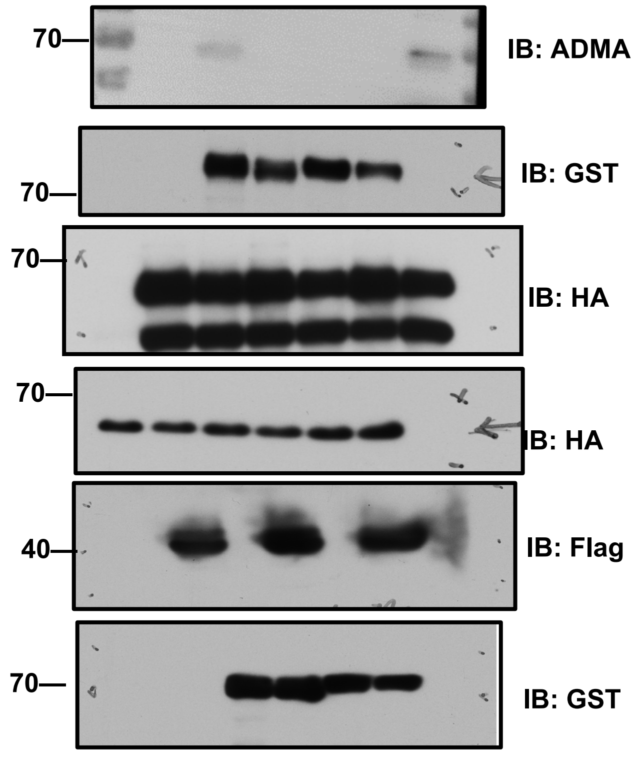

Figure 6g

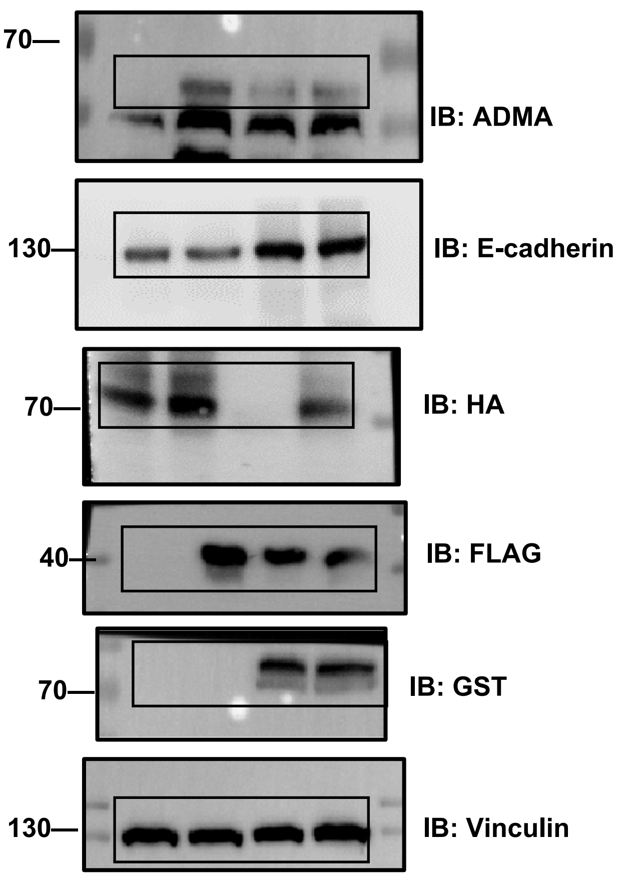

Figure 6h

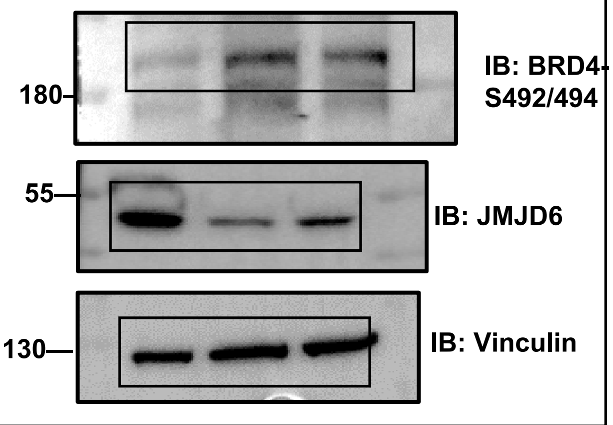

Figure 6i

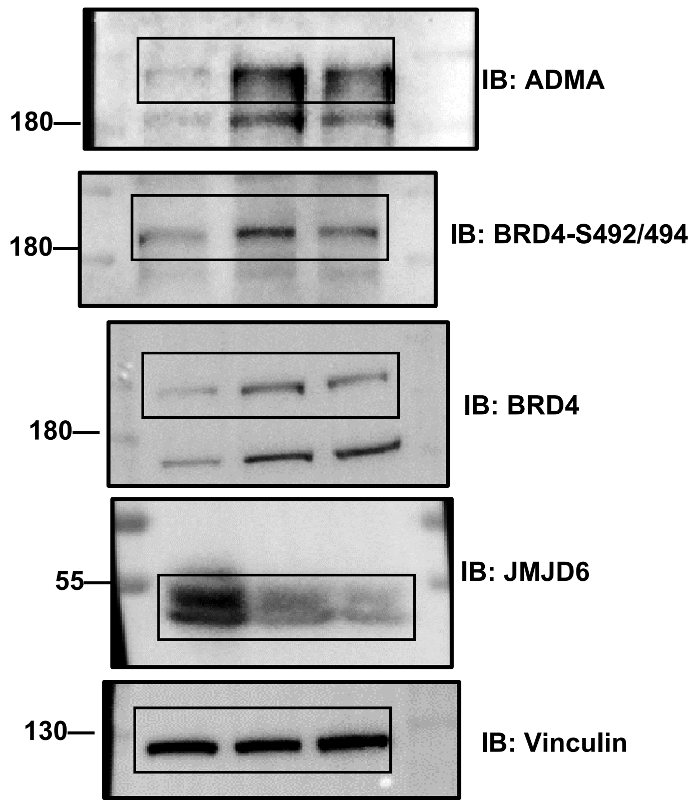

Figure 7a

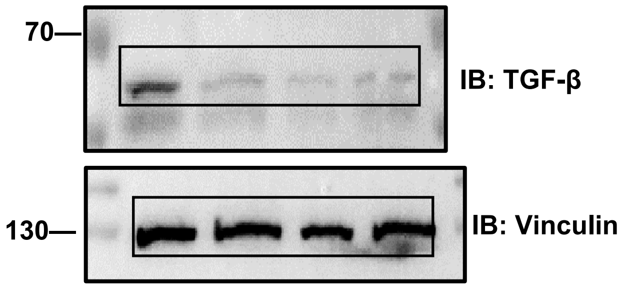

Figure 7b

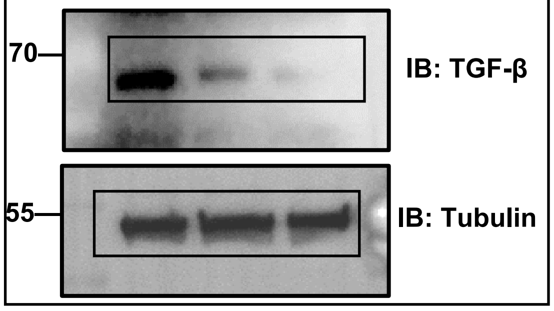

Figure 7c

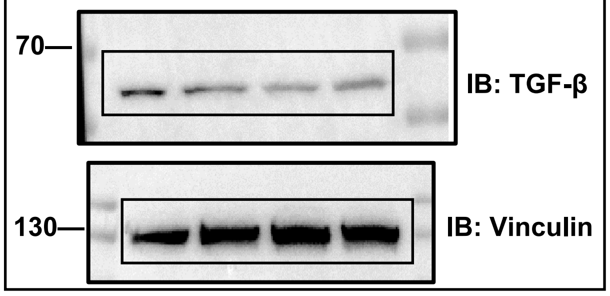

Figure 7d

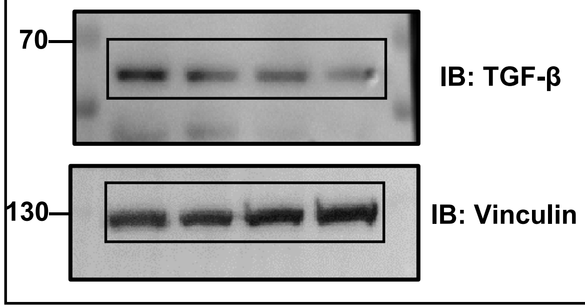

Figure 7e

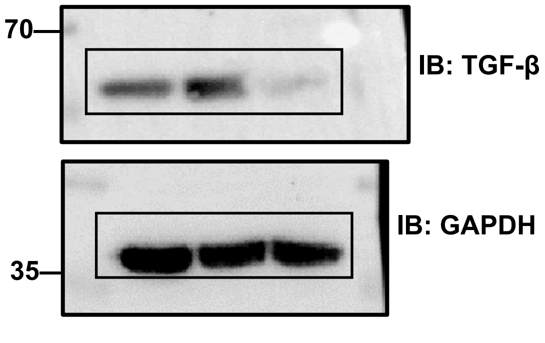

Figure 7f

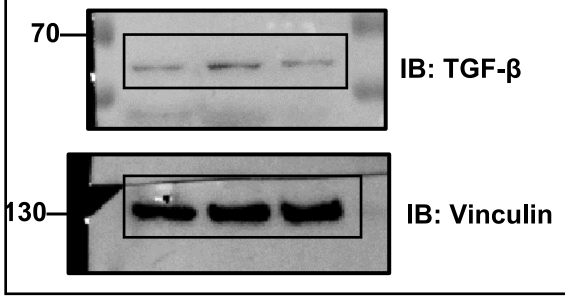

Figure 7g

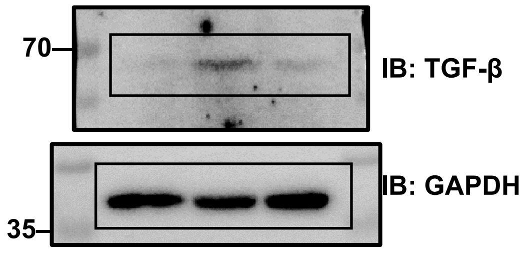

Figure 7h

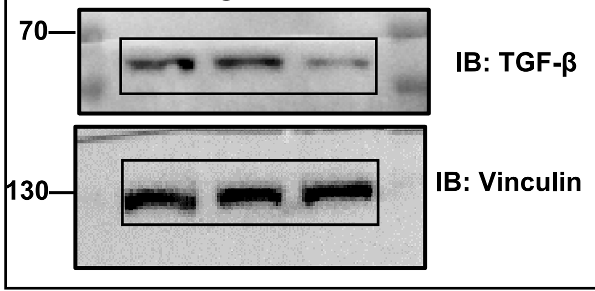

Figure 7i

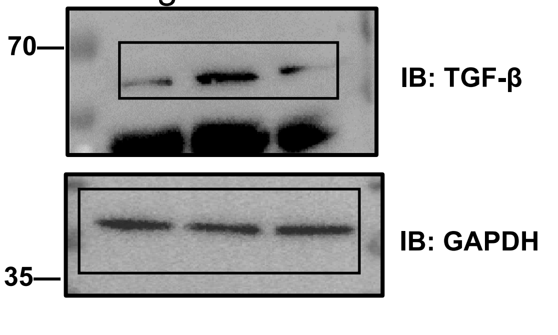

Figure 7j

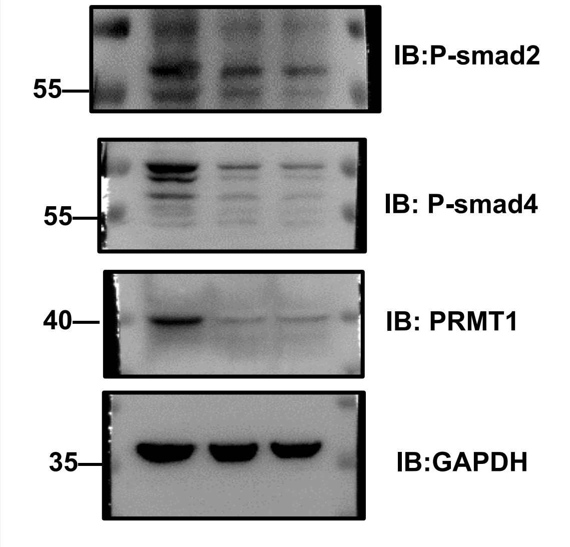

Figure 7k

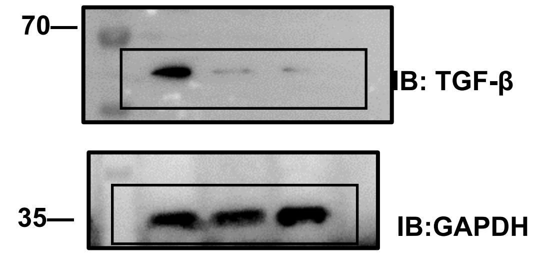

Figure S1c

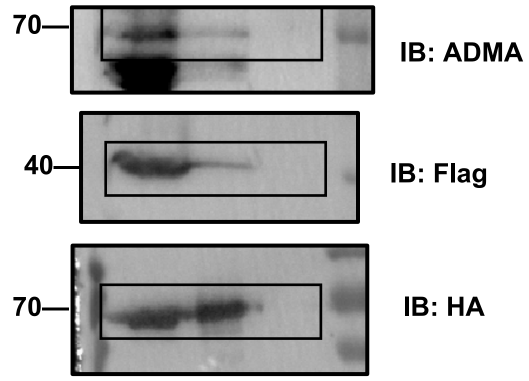

Figure S1d

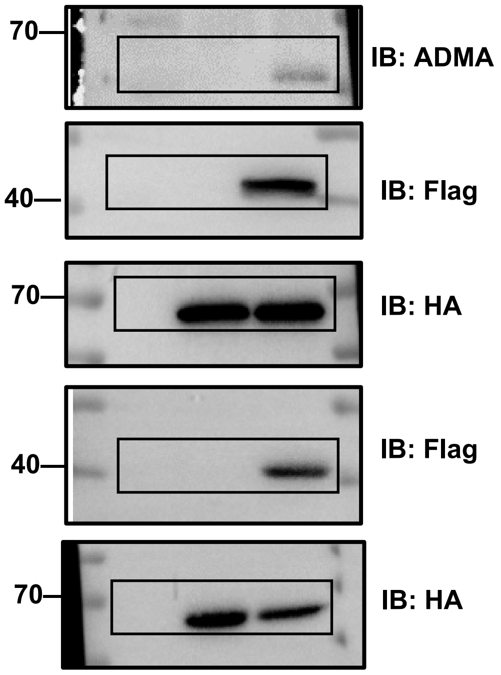

Figure S1e

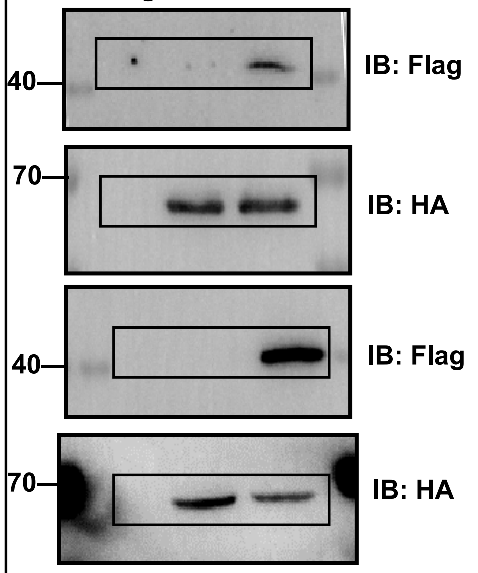

Figure S1f

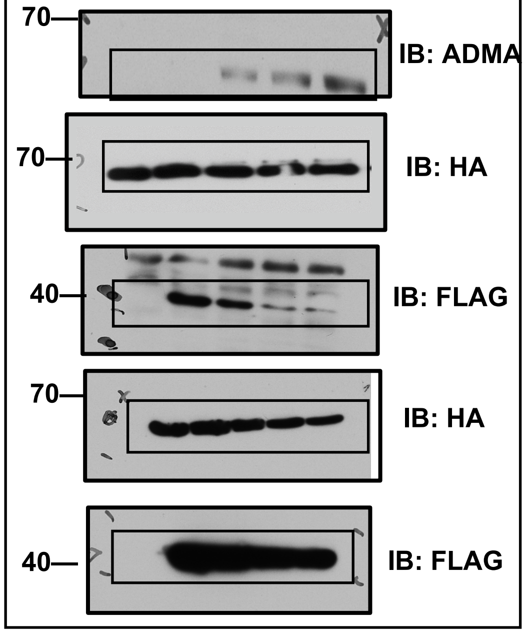

Figure S2a

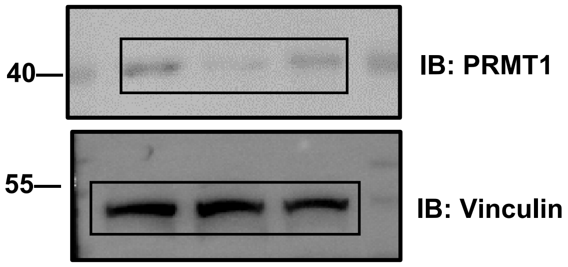

Figure S3a

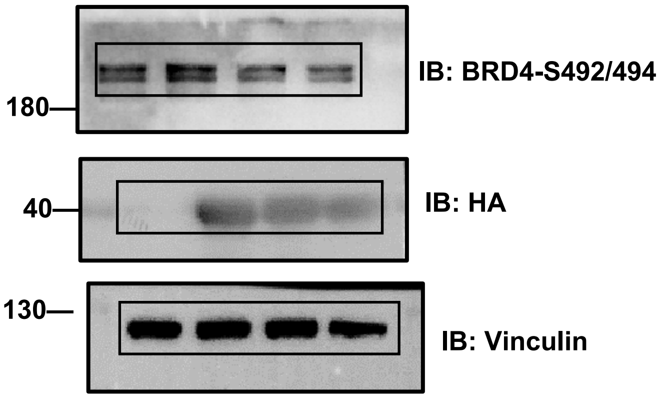

Figure S3b

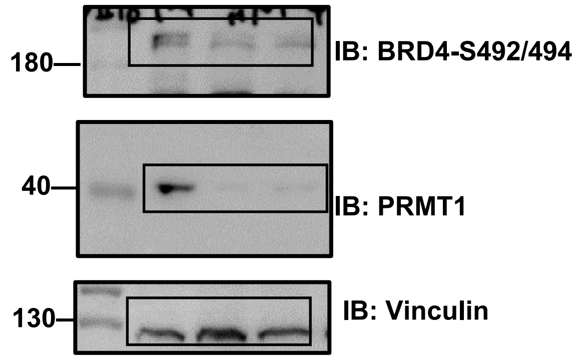

Figure S3c

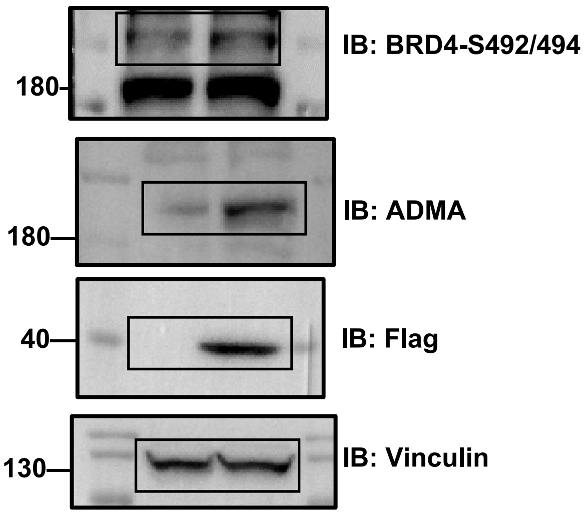

Figure S3d

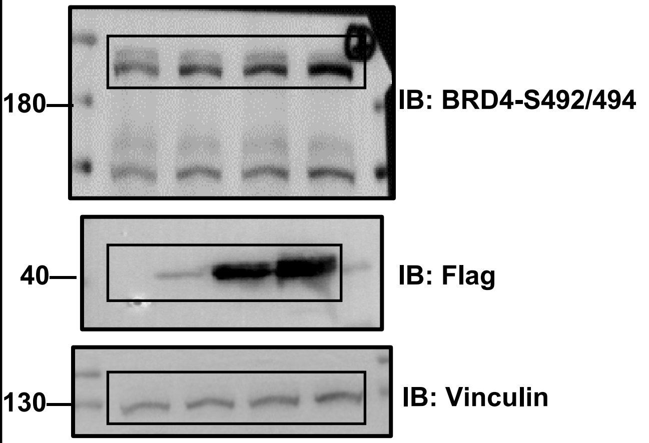

Figure S3e

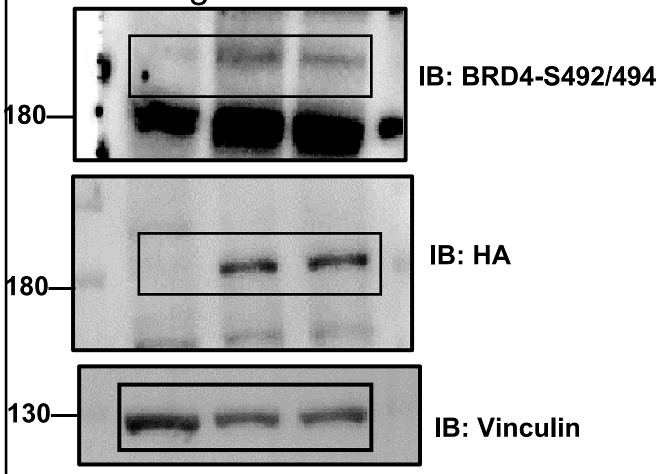

Figure S3f

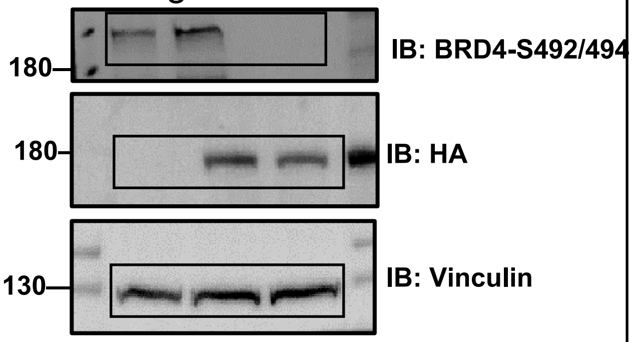

Figure S4a

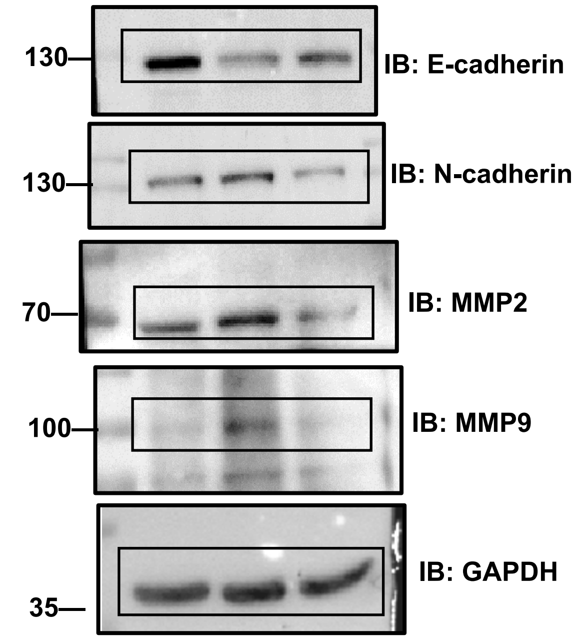

Figure S4b

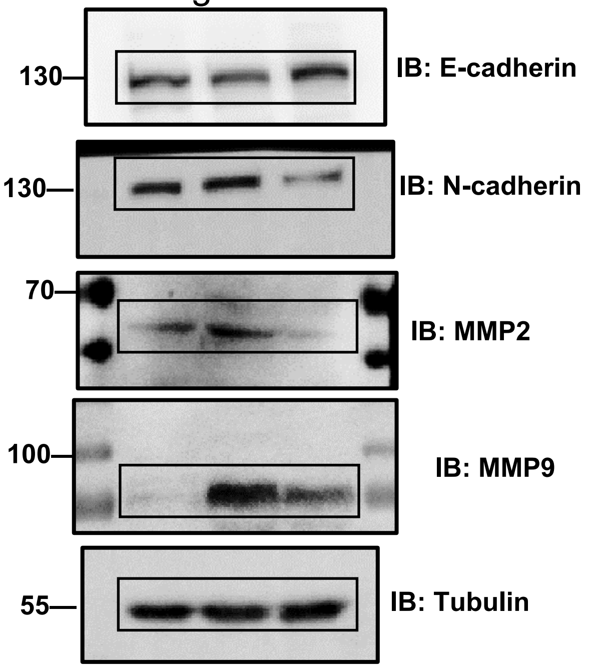

Supplement: Supplementary file 8 — WB original images [file 41419_2023_6149_MOESM8_ESM.pdf]
